# Targeted LC-ESI-MS^**2**^ characterization of human milk oligosaccharide diversity at 6 to 16 weeks post-partum reveals clear staging effects and distinctive milk groups

**DOI:** 10.1007/s00216-020-02819-x

**Published:** 2020-08-14

**Authors:** Marko Mank, Hans Hauner, Albert J. R. Heck, Bernd Stahl

**Affiliations:** 1 Danone Nutricia Research, Uppsalalaan 12, 3584 CT Utrecht, The Netherlands; 2 Else Kröner-Fresenius Center for Nutritional Medicine, Klinikum rechts der Isar, Technische Universität München, Ismaninger Straße 22, 81675 Munich, Germany; 3 Nutritional Medicine Unit, Research Center for Nutrition and Food Sciences (ZIEL), Technische Universität München, Weihenstephaner Berg 1, 85354 Freising, Germany; 4 Biomolecular Mass Spectrometry and Proteomics, Bijvoet Center for Biomolecular Research and Utrecht Institute for Pharmaceutical Sciences, University of Utrecht, Padualaan 8, 3584 CH Utrecht, The Netherlands; 5 Netherlands Proteomics Center, Padualaan 8, 3584 CH Utrecht, The Netherlands; 6 Utrecht Institute for Pharmaceutical Sciences, Utrecht University, Universiteitsweg 99, 3584 CG Utrecht, The Netherlands

**Keywords:** Human milk oligosaccharides (HMOs), Stages of lactation, Human milk groups/milktyping, Targeted LC-MS^2^, Principal component analysis (PCA), Secretor (Se) and Lewis (Le) gene–dependent or Secretor (Se) and Lewis (Le) gene–independent HMOs, 3-FL, 6′-SL

## Abstract

Many molecular components in human milk (HM), such as human milk oligosaccharides (HMOs), assist in the healthy development of infants. It has been hypothesized that the functional benefits of HM may be highly dependent on the abundance and individual fine structures of contained HMOs and that distinctive HM groups can be defined by their HMO profiles. However, the structural diversity and abundances of individual HMOs may also vary between milk donors and at different stages of lactations. Improvements in efficiency and selectivity of quantitative HMO analysis are essential to further expand our understanding about the impact of HMO variations on healthy early life development. Hence, we applied here a targeted, highly selective, and semi-quantitative LC-ESI-MS^2^ approach by analyzing 2 × 30 mature human milk samples collected at 6 and 16 weeks post-partum. The analytical approach covered the most abundant HMOs up to hexasaccharides and, for the first time, also assigned blood group A and B tetrasaccharides. Principal component analysis (PCA) was employed and allowed for automatic grouping and assignment of human milk samples to four human milk groups which are related to the maternal Secretor (Se) and Lewis (Le) genotypes. We found that HMO diversity varied significantly between these four HM groups. Variations were driven by HMOs being either dependent or independent of maternal genetic Se and Le status. We found preliminary evidence for an additional HM subgroup within the Se- and Le-positive HM group I. Furthermore, the abundances of 6 distinct HMO structures (including 6′-SL and 3-FL) changed significantly with progression of lactation.

**Electronic supplementary material:**

The online version of this article (10.1007/s00216-020-02819-x) contains supplementary material, which is available to authorized users.

**Figure:**
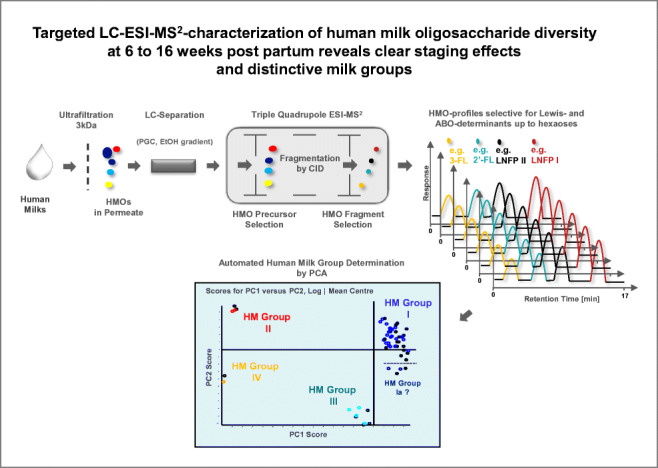
Graphical abstract

## Introduction

Human milk (HM) is largely defined by the abundance of 5 major constituents comprising lactose, lipids, oligosaccharides, proteins, and water. Published values for lactose, lipid, and protein concentrations in mature milk 1–2 months post-partum (pp) are 67–78 g/l, 32–36 g/l, and 9–12 g/l [1] respectively. Even broader ranges in concentration of HM components apply if also colostral and transitional milks (5–14 days pp) are considered [2]. The reported concentrations for total HMOs in mature milk range between ~ 3 and 18 g/l [3–8]. Until now, more than 150 HMO structures have been revealed and confirmed by various analytical approaches including HPLC, mass spectrometry, and NMR to name a few [9–13]. Abundant individual HMOs like 2′-fucosyllactose (2′-FL), lacto-N-tetraose (LNT), lacto-N-neotetraose (LNnT), lacto-N-fucopentaose I (LNFP I), difucosyl-lacto-N-hexaose II (DF-LNH II), trifucosyl-lacto-N-hexaose (TF-LNH), 6′-sialyllactose (6′-SL), and disialyl-lacto-N-tetraose(DSLNT) may be present in the low gram per liter range [3]. This set of known HMO structures represents only a few of more than 1000 compositional or structural variants as anticipated from MALDI-MS data of high molecular weight HMO fractions with molecular masses > 8 kDa. The latter mass range translates into a degree of polymerization (DP) for particular HMOs > 43 [4].

Additional variations in human milk oligosaccharide profiles can be introduced by preterm delivery (lower lactose concentrations compared with term birth [14]) or are observed at different stages of lactation. In fact, literature reports an overall decrease of HMOs [15–17] over time during stages of lactation and the highest concentration of approximately 20 g/l total HMOs for term delivered infants is described to appear on around day 4 post-partum [18]. Staging effects are also observed for other mammalian milks like bovine or caprine milk [19–22]. Finally, also regional differences may have an influence on HMO variations exemplified by 100% presence of 2′-FL in milks of 156 Mexican women versus only 46% presence in milks of 26 Philippine donors [23]. Beyond their presence in human milk, HMOs have also been found in blood samples of both, mothers (gestational week 10–35) and breastfed offspring [24–28] and are even found in the amniotic fluid [29].

Bovine milk which is the basis for many infant milk formulations contains on average less lactose (approximately 40–50 g/l lactose [30]) and only minute amounts of bovine milk oligosaccharides (BMOs). BMOs comprise about 40 structures [31]. Among these BMOs, 3′-sialylactose and 6′-sialylactose are prevalent, but also galactosyllactoses (Gal-Lacs) may be of interest. As reported by Nakamura et al. in 2003 [20], sialyllactose concentrations in bovine milk samples from 3 days post-partum range between 0.1 g/L for 3′-SL and < 0.05 g/L for 6′-SL. Mature bovine milk contains approximately 0.03–0.12 g/L 3′-SL and 0.03–0.09 g/L 6′-SL [32] which is below the mean concentrations found in term human milk for 3′-SL (0.16 g/L) and 6′-SL (0.6 g/L) [3].

**Fig. 1:**
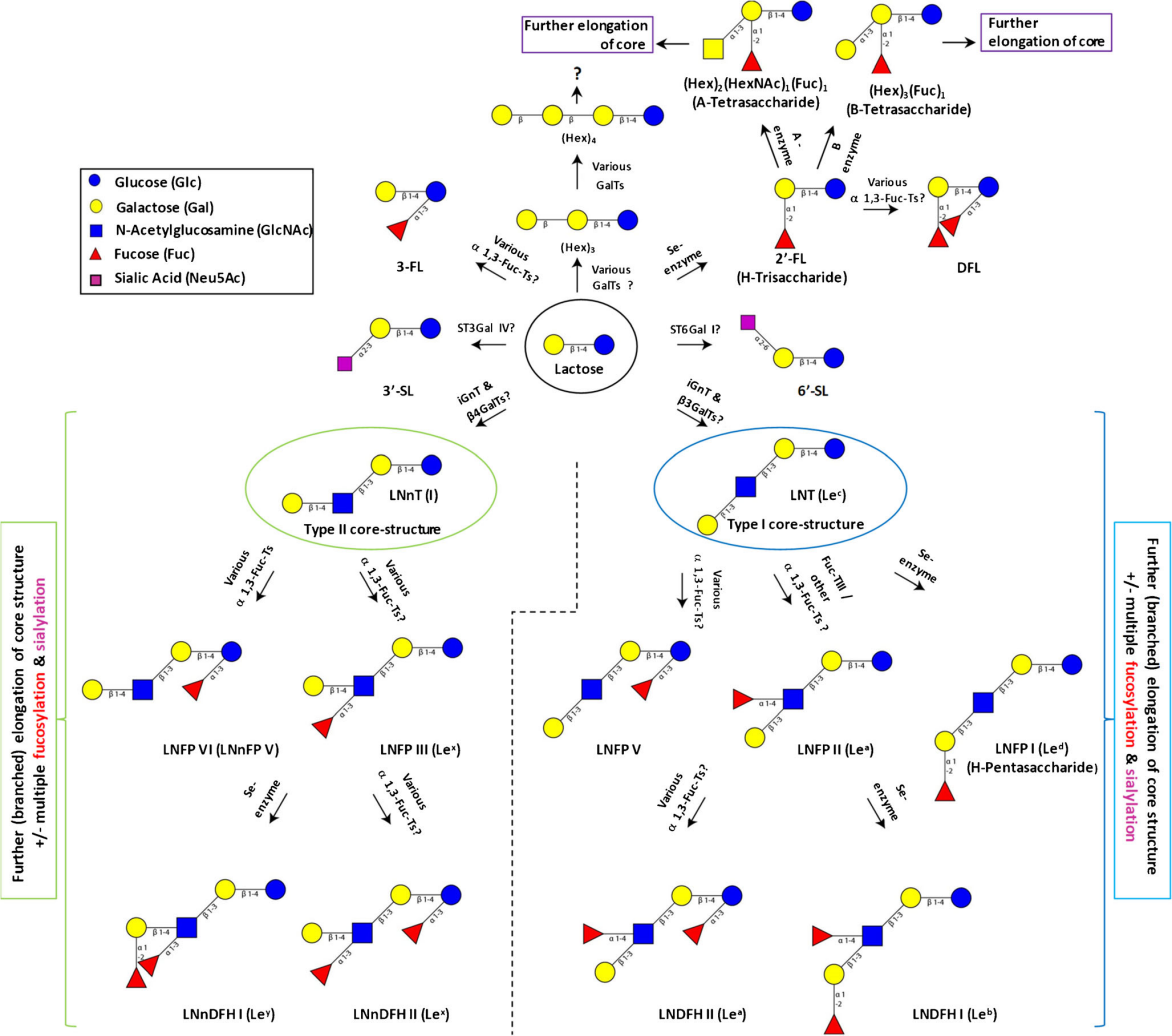
Major HM HMO structures and related glycosyltransferases [9, 33–36] involved in their mammary gland biosynthesis starting from lactose as a precursor. Certain HMOs resemble ABH [37] or Se/Le system–related antigens [38, 39]. 3-Fucosyllactose (3′-FL), 2′-fucosyllactose (2′-FL), 3′-sialyllactose (3′-SL), 6′-sialyllactose (6′-SL), difucosyllactose (DFL), lacto-N-tetraose (LNT); lacto-N-neotetraose (LNnT); tentative blood group B tetrasaccharide (Hex)_3_(Fuc)_1_; tentative blood group A tetrasaccharide (Hex)_2_(HexNAc)_1_(Fuc)_1_; lacto-N-fucopentaose I (LNFP I); lacto-N-fucopentaose II (LNFP II); lacto-N-fucopentaose III (LNFP III); lacto-N-fucopentaose V (LNFP V), lacto-N-fucopentose VI (LNFP VI = LNnFP V), lacto-N-neodifucohexaose I (LNnDFH I), lacto-N-neodifucohexaose II (LNnDFH II), lacto-N-difucohexaose I (LNDFH I), lacto-N-difucohexaose II (LNDFH II); monosaccharide symbols and structural representations of oligosaccharides were drawn with GlycoWorkbench [40] according to CFG proposals [41]

Biosynthesis of HMOs is governed by a limited set of glycosyltransferases present in the mammary gland and human milk itself [12]. Therefore, with only a few exceptions, the structural arrangement of most human milk oligosaccharides can be described by the following general scheme (see also Fig. 1): The sequence of each HMO molecule starts with a lactose building block (Galβ1–4Glc) introducing a reducing end which is a common feature of all known HMOs described until now. This lactose building block can be extended with a dimeric residue consisting of galactose and *N*-actetylglucosamine (Gal-GlcNAc). As suggested by Kobata et al. 2010 [42], the transfer of Gal-GlcNAc to the terminal Gal of lactose may be accomplished by consecutive action of either β-1,3-*N*-acetylglucosaminyltransferase (iGnT) [43] and β1,4-galactosyltransferases (β4GalTs) [44] or iGnT and β1,3-galactosyltransferases (β3GalTs) [45]. Thereby, two basic types of HMO core structures can be created: a type I core (Galβ1–3GlcNacβ1–3Galβ1–4Glc, LNT) or a type II core molecule (Galβ1–4GlcNacβ1–3Galβ1–4Glc, LNnT). In HM, type I core HMOs are predominant over type II structures [46]. This feature distinguishes HM from other species including apes and monkeys. According to Urashima et al. [46], one possible explanation for this phenomenon is the role of type I oligosaccharides as important substrates for beneficial bifidobacteria. Moreover, the two types of neutral core entities (i.e., LNT or LNnT) may be further extended by successive addition of more Gal-GlcNAc blocks. If Gal-GlcNAc residues are appended to the core structures via β1,3-glycosidic linkage, no further elongation will occur after this Gal-GlcNAc block. If, in contrast, Gal-GlcNAc dimers are added via action of β6N-acetylglucosaminyltransferase (IGNT) [47] through β1,6-glycosidic linkages, branching will occur and further elongation of the β1,6-glycosidically linked branches is possible. Thereby, up to 19 complex HMO core structures can be created [38]. Finally, these core structures or lactose itself may be furthermore decorated by additional fucoses (Fuc), and sialic acid (Neu5Ac) moieties through human fucosyl-[33] or sialyl-transferases [34]. However, it remains to be explored which of the known human sialyl- and fucosyltranferases are actually actively involved in the mammary gland biosynthesis of HMOs. More certainty already exists with respect to HMO fucosylation exerted by the Se-enzyme [35] and fucosyltransferase 3 [48] as explained in the next paragraph. Finally, exceptions from the general HMO building scheme outlined above may exist and are e.g. exemplified by the presence of galactosyllactoses (GL) in human milk. Here, only a single Gal is added to lactose. Literature describes several galactosyllactose isomers such as 6′-GL, 3′-GL, and 4′-GL. To date, information about GL concentrations in HM is still rare, but first published levels are in the milligram per liter range [49].

Inherited maternal genetic predispositions may induce remarkable inter-individual differences in oligosaccharide synthesis and resulting individual HMO profiles. For instance, Secretor (Se) and Lewis (Le) genes on chromosome 19 encode for two different fucosyltranferases: the Se-enzyme and fucosyltransferase 3 (Fuc-TIII) [33]. The Se-enzyme is expressed in the case of an active Se gene (FUT 2) and Fuc-TIII in the case of an active Le gene (FUT 3). The Se-enzyme specifically appends fucose (Fuc) via α1,2-glycosidic linkage to type 1 HMOs (e.g., lacto-N-tetraose (LNT)) or to type 2 HMOs (e.g., lacto-N neotetraose (LNnT)), whereas in contrast Fuc-TIII catalyzes the formation of α1,3/4-fucosylated HMOs. Primarily catalyzed by the Se-enzyme and Fuc-TIII, 4 different ways of HMO-fucosylation at distinct monosaccharides and distinct sites within type I or II core HMO structures can occur [12]: α1,2-linkage to non-reducing end Gal, α1,3- or α1,4-linkage to *N*-acetylglucosamin (GlcNAc), or α1,3-linkage to reducing end Glc. Neutral or sialylated acidic HMOs with core structures smaller or larger than tetraoses can be decorated in the same way and lead to a multitude of known or hypothetically possible fucosylated HMO structures.

Figure 1 schematically exemplifies fucosylation of LNT (resembling a Lec-antigen [39]) and LNnT (Le I-antigen) by activity of Se-enzyme, FucTIII, or further α1,3-fucosyltransferases [50]. Resulting HMOs are e.g. lacto-N-fucopentaoses (LNFPs) such as LNFP I, II, III, and V. Such fucosylated HMOs can resemble ABO- or Se/Le system–related antigens like the Le^d^-antigen in case of LNFP I, the Le^a^-antigen in case of LNFP II, or the Le^x^-antigen in case of LNFP III [37, 39]. Additionally, LNFP VI (the type II core variant of LNFP V) may be formed, too. It was recently detected in human milk by Bao et al. [51] using negative ion mode LC-MS after conversion of HMOs into alditols. Other products of the Se-enzyme, Fuc-TIII (or other α1,3-fucosyltransferases) are human milk oligosaccharides like fucosyllactoses (2′FL or 3-FL), difucosyllactose (DFL), the lacto-N-difucohexaoses LNDFH I (Le^b^-antigen), LNnDFHI (Le^Y^-antigen), LNDFH II and LNnDFH II, or higher molecular weight neutral and acidic HMOs with either linear or branched core structures.

Based on the presence, absence, or concomitance of α1,2- and α1,3/4-fucosylated HMOs like LNFP I and LNFP II in human milk, 4 different human milk groups (groups I, II, III, and IV) are currently distinguished [52, 53]. However, just recently, group I milks from Chinese donor were further subdivided into 2 new subgroups based on significantly different levels in e.g. 2′-FL, DFL, LNT, LNFP I, or F-LNO [54]. Assignment of human milk samples to one of the 4 main human milk groups I–IV can e.g. be performed by ESI-LC-MS or HPAEC HMO analysis and subsequent evaluation e.g. of the respective LNFP I and LNFP II patterns. Moreover, corresponding Le and Se gene product–dependent glycan epitopes may also be found in maternal blood, i.e., on erythrocytes or glycoproteins like mucins [55]. As mentioned before, other α1,3-fucosyltransferases such as plasma α-1,3-fucosyltransferase (E.C. 2.4.1.152), different from Se-enzyme or Fuc-TIII and independent from the Le blood group system, may be active in human milk donors as well [39, 56, 57].

Table 1 summarizes the interdependence of maternal Lewis and Secretor genotypes, encoded fucosyltransferases, Lewis blood group phenotypes, related carbohydrate epitopes, resulting milk groups, and some prevalent HMO structures found in specific milk groups.

**Table 1:** Interdependence of maternal Lewis and Secretor genotypes, encoded fucosyltransferases (Fuc-Ts), Lewis blood groups, resulting carbohydrate epitopes, HMO structures, and milk groups. *2′-FL*, 2′-fucosyllactose; *LDFT*, lacto-Di-fucotetraose; *LNFP I*, lacto-N-fucopentaose I; *LNFP II*, lacto-N-fucopentaose II; *LNFP III*, lacto-N-fucopentaose III; *LNFP V*, lacto-N-fucopentaose V; *LNDFH I*, lacto-N-difucohexaose I; *LNDFH II*, lacto-N-difucohexaose II. Adapted from Oriol et al. 1986, Thurl et al. 1997, and Blank et al. 2011

Genotypes		Encoded fucosyl-transferases/gene loci	Resulting phenotypes			Human milk group	Prominent neutral HMO structures up to hexaoses^a^
			Erythrocytes		Human milk		
			Lewis	Prevalence in France [39]	Fucosylated carbohydrate epitopes		
Secretor	Lewis						
Se/−	Le/−	Se-enzyme/FUT2, Fuc-TIII/FUT3	a-b+c-d-	69	Fuc (α1-2)R, Fuc (α1-4)R, Fuc (α1-3)^c^R	**I**	**2′FL,** 3′-FL, **DFL,** LNT, LNnT, **LNFP I, LNFP II,** LNFPIII, **LNDFH I, LNDFH II**
se/se	Le/−	Fuc-T III/FUT3	a+b-c-d-	20	Fuc (α1-4)R, Fuc (α1-3)^c^R	**II**	3′-FL, LNT, LNnT, **LNFP II**, LNFPIII, **LNDFH II;** (*2′FL, DFL, LNFP I, LNDFH I*)
Se/−	le/le	Se-enzyme/FUT2	a-b-c-d+	9	Fuc (α1-2)R, Fuc (α1-3)^c^R	**III**	**2′FL**, 3′-FL, **DFL**, LNT, LNnT**, LNFP I**, LNFPIII; (*LNFP II, LNDFH I, LNDFH II*)
se/se	le/le	Le- and Se- independent Fuc-Ts (e.g., Fuc-TVI/FUT6 [57]	a-b-c+^b^d-	1	Fuc (α1-3)^c^R	**IV**	3′-FL, LNT, LNnT, LNFPIII; (*2′FL, DFL, LNFP I, LNFP II, LNDFH I, LNDFH II*)

^a^Bold black letters indicate major HMO structures present in human milks due to active Le or Se genes; italic letters in parentheses indicate HMO structures lacking or being present in very low concentrations in human milk due to fully or partly inactive Le or Se genes; regular black letters indicate prominent HMOs occurring in HM independent from maternal Le/Se activity

^b^According to Hanfland et al. 1986 [58], there may be more than one Le^c^ antigenic determinant: Le^c^ epitopes may comprise non-fucosylated type I or type II glycan residues or extended unbranched or branched glycan residues with α1-3-fucosylation at the penultimate GlcNAc positioned before the non-reducing end of the glycan chain

^c^Fuc (α1-3-)R epitope can also be catalyzed by Le/Se-independent FUT, e.g., FUT 6 [57]

HMOs confer many intriguing functional properties of which some are likely to facilitate or influence healthy development of neonates [59–63]. Many of the published prebiotic, anti-infective, or immunomodulatory properties are highly structure specific [64–70]. They may vary between HMOs of the same monosaccharide composition, but different core types or fucosylation sites like LNFP I, LNFP II, or LNFP III. Also blood group A and B carbohydrate determinants can be present as free carbohydrates in HM [71]. Together with galactosyllactoses, they deviate from the HMO building scheme described above. Nevertheless, they may bear anti-infective potential relevant for early life and beyond, e.g., by blocking the cholera toxin (CT) subunit B (CTB) of *Vibrio cholerae* from adhesion to mucins and intestinal epithelial cells of the small intestine [72].

As outlined above, different structure-effect relationships could be attributed to HMOs which are isomers but share the same monosaccharide composition. Thus, development and application of hyphenated analytical methods like LC-MS which can distinguish such subtle structural HMO features may be key to decipher still unknown (beneficial) biological functions of HMOs and other glycans. To accelerate the analysis of larger sample numbers available from e.g. human milk cohorts or clinical studies, analytical approaches with short run times and avoidance of tedious sample pre-treatment would be of advantage. In turn, this will help to improve statistical association of distinct HMO patterns with related health benefits for infants. In addition, (semi-)quantitative assays should be preferred over mere qualitative ones to allow for elucidation of concentration-dependent biological HMO effects.

Therefore, in this work, we applied a recently developed targeted semi-quantitative LC-ESI-MS^2^ approach with high structural selectivity [73], easy sample preparation, and comparatively low run times of ~ 18 min per sample to 2 × 30 human milk specimen. These specimens were derived from a control group of the INFAT study which was not subjected to nutritional intervention. The used sample set comprised human milks collected at 6 and 16 weeks post-partum to elicit possible changes in HMO profiles between those 2 different stages of lactation.

Principal component analysis (PCA) was employed as a convenient and efficient strategy for mining of structural HMO information inherent in the acquired multiple reaction monitoring (MRM) LC-ESI-MS^2^ data sets.

## Experimental section

### Human milk samples from INFAT study with unknown milk group and HMO status

A subset of 60 human milk samples derived from 30 healthy women resident in southern Germany and enrolled in a dietary intervention study called the INFAT study [74, 75] was subjected to MRM LC-ESI-MS^2^ analysis. Every woman donated 2 milk samples at 6 and 16 weeks post-partum. Written consent from the donors was provided prior to analysis and approval was given by the ethical committee of the Faculty of Medicine of the Technical University of Munich, Germany (project number 1479/06 on 14 September 2012). The human milk samples used for this LC-MS investigation were derived from a control group not subjected to dietary intervention. Samples were obtained as described elsewhere [74]. Briefly, maternal breast milk samples were collected in the study center in the morning after an overnight fast in a standardized manner using a breast pump, aliquoted, and immediately stored at − 86 °C until analysis. Samples were thawed once to take aliquots for different kinds of analysis and stored again at − 20 °C until further usage for negative ion mode MRM LC-MS^2^ analysis.

### Chemicals, reagents, and disposables

Ammonium acetate (p.a.), acetonitrile (LC-MS grade), and ethanol (gradient grade for HPLC) were from Merck, Darmstadt, Germany. LC-MS grade H_2_O (HiPerSolv Chromanorm) was from Prolabo, VWR International, Darmstadt, Germany. Alternatively, ultrapure lab water, 18 kΩ, < 5 ppm TOC produced by a MilliQ Advantage Ultrapure Water System from Merck Millipore (Darmstadt, Germany) was used. Clear glass 300-μl micro LC-MS vials were from Thermo Fisher Scientific (Waltham, MA, USA, #60180-507). Micro vial screw caps with pre-slit PTF/silicone membrane were from Thermo Scientific (Langerwehe, Germany). Two-milliliter polypropylene LoBind® test tubes were from Eppendorf AG (Hamburg, Germany, #022431102). Five hundred-microliter volume Amicon Ultra centrifugal filter devices with 3-kDa cutoff were from Millipore (Billerica, MA, USA).

### Preparation of human milk samples and MRM LC-ESI-MS^*2*^ analyses of HMOs

Pre-treatment of human milk samples, targeted semi-quantitative negative ion MRM LC-ESI-MS^2^ analyses of HMOs, software settings, evaluation of data, and manual assignment of human milk groups were essentially conducted as recently published in more detail by Mank et al. [73]. Briefly, preparation of HM milk samples was accomplished by adding 15 μl of α-l-arabinopentaose internal standard (ITS) solution (0.05 mmol/l) to 135 μl of human milk. Frozen human milk samples had been thawed before at room temperature and were vortexed well. The combined sample-ITS-solution was further diluted 1:11 (v/v) in 2-ml Eppendorf test tubes by adding 1350 μl H_2_O (LC-MS grade). Four hundred fifty microliters of the diluted mixture was transferred into a previously washed 500-μl Amicon Ultra centrifugal filter device with 3-kDa cutoff. Ultrafiltration (UF) was performed at 14,000*g* for 1 h. The UF permeate was transferred into a 300-μl glass LC-MS vial with screw top (Thermo Fisher Scientific, Waltham, MA, USA) and either stored at − 20 °C until further use or directly subjected to LC-MS analysis. Sample preparation steps were conducted at room temperature.

For negative ion mode multiple reaction monitoring LC-ESI-MS^2^ analyses, a 1100 series HPLC stack (Agilent, Santa Clara, CA, USA) with a solvent rack, degasser, binary solvent pump, high-performance autosampler, column oven, and DAD detector was coupled with a 3200 QTRAP triple quadrupole linear ion trap MS instrument (Sciex, Framingham, MA, USA). A Hypercarb 2.1 × 30 mm porous graphitized carbon (PGC) column (5-μm particle diameter) following a 2.1 × 10 mm Hypercarb pre-column (both Thermo Fisher Scientific, Waltham, MA USA) served as the stationary phase. Column temperature was kept at 45 °C, and sample temperature in the autosampler was 22 °C. The DAD detector was used to monitor possible peptide or protein contaminations at 215- and 280-nm wavelengths. Five-microliter sample solutions prepared as described above were injected into the LC-MS system. Carry over was minimized by flushing the sample needle externally for 5 s with 20% aqueous isopropanol after each injection and by running a water and isopropanol blank between each HM sample. Elution of HMOs was achieved with a ~ 18 min H_2_O-EtoH gradient at a flow rate of 400 μl/min (see Table S1 in Electronic Supplementary Material, ESM). The solvents used to form the gradient were A (5 mM aqueous NH_4_CH_3_COO) and B (80% EtOH (v/v) with 5 mM aqueous NH_4_CH_3_COO). Analyst 1.4 software (Sciex, Framingham, USA) was used to control the complete LC-MS configuration and for further evaluation of LC-MS data.

Using this LC-MS platform, a first set of 10 distinct, specific, and partly isomeric HMO structures could be integrated into the targeted MRM LC-MS^2^ method. HMO-specific MRM transitions were based on monoisotopic molecular masses of deprotonated precursor ions and respective diagnostic fragment ions. Unit resolution was chosen to conduct MRM analyses. MRM transitions were continuously monitored in negative ion mode during the entire MRM LC-MS^2^ gradient elution (see [73]). One LC-MS run took approximately 18 min. HMO structures which were determined by this first set of (isomer-) specific MRM transitions included 2′-FL, 3-FL, 3′-SL, 6′-SL, DFL, LNT, LNnT, LNFP I, LNFP II, and LNFP III (see Table S2 in ESM). The correct identification of the latter HMO structures via their respective MRM transitions was proven by analysis of pure HMO standards (concentrations ~ 0.03 to 0.05 mg/ml) which were run together with the INFAT human milk study samples. The used pure HMO standards had been isolated from pooled human milk and were identical to those already described in more detail by Mank et al. 2019 [73].

Furthermore, a second set of 11 additional MRM transitions representing further OS structures could be included into the same LC-MS^2^ approach as mentioned before (see Table S3 in ESM). Compared with the first MRM set, HMO monosaccharide compositions rather than exact structural features could be directly deduced from the fragment ions used in these MRM transitions (see [73]). Therefore, either one HMO composition or up to two different structures were assigned per MRM transition in set 2. Compounds which could be characterized by this 2nd set of MRM transitions were (Hex)_2_ or lactose, (Hex)_3_ or galactosyllactose, sialyllactose or (Hex)_2_ (NeuAc)_1_, (Hex)_4_, α-arabinopentaose (internal standard), tentative blood group B-tetrasaccharide or (Hex)_3_(Fuc)_1_, tentative blood group A-tetrasaccharide or (Hex)_2_(HexNAc)_1_(Fuc)_1_, LNFP V or LNFP VI, LNDFH I or LnNDFH I, and LNDFH II or LNnDFH II. Despite the more putative assignment of the latter HMOs, at least LN(n)DFH I could also be distinguished from LN(n) DFH II due to a retention time delta of approximately 2 min as shown in Mank et al. 2019 [73]. All 60 human milk samples derived from the INFAT study were analyzed by the MRM LC-ESI-MS^2^ approach described above in a randomized and anonymized manner (for exemplary MRM LC-ESI-MS^2^ traces of some pure HMO standards (LNFPI, II, III, LNT, and LNnT) and of abundant HMOs in a HM study sample, please see Fig. S1a)–f) in ESM).

If not otherwise mentioned, the settings for identification of HMOs, peak integration, and relative quantitation of HMOs using negative ion mode MRM LC-ESI-MS have been applied as described by Mank et al. [73]. Also, run to run variations for individual HMOs were determined as described in this reference by 10 repetitive injections of the same HM sample. Results were expressed as individual relative standard deviations (RSDs) for distinct HMOs: The resulting RSDs were less than 12% for LNFP I, LNFP II, LNFP III, LNT, and LNnT, up to 20% for total SL, (Hex)_3_, 3′-SL, 6′-SL, and (Hex)_2_/Lac, and maximally 38% for all other targeted HMOs. After integration of peak areas, dimensionless responses *R* were calculated for each HMO peak in a MRM trace by dividing its area under the curve by the area under the curve yielded for the internal standard α-arabinopentaose. The limit of detection (LOD) for individual HMOs was defined by a signal to noise (*S*/*N*) ratio of approximately 3:1 [76]. Peaks below LOD were not considered and reported as zero values.

For further processing and evaluation, original LC-MS raw data sets were exported from Analyst 1.4 software to Microsoft Excel version 14.0.7145.5000 (32 bit), Sciex Markerview version 1.2.1., or IBM SPSS version 19. Basic statistics (*t* test, Welsch’s *t* test) were carried out within Markerview. Further statistical tests (see below) were accomplished with SPSS.

### Manual assignment of HM samples to HM groups

Manual assignment of human milk INFAT study samples to one of the four commonly known human milk groups was based on the absence, presence, or concomitance of LNFP I and LNFP II. Biosynthesis of these two HMOs and other HMOs is governed by the maternal Se/Le status (see Table 1). If both LNFP I and LNFP II were detected in a sample, human milk group I was assigned. If LNFP I was absent but LNFP II was present, milk group II was assigned. If only LFNP I was detected but not LNFP II, group III was assigned and if neither LNFP I nor LNFP II could be detected, then milk group IV was assigned. Peaks were considered for evaluation if the detected responses *R* exceeded the value of 0.2. The distribution of HM samples across human milk groups I, II, III, and IV was expressed in percentages and graphically visualized using SPSS software.

### Automated HM group determination by principal component analyses (PCA) and statistical evaluation of lactation and HM group–specific HMO features

To relief future milk grouping attempts from tedious, time-consuming manual inspection of analytical LC-MS raw data, principal component analysis (PCA) was used as a means to automatically recognize different milk groups. Dedicated software (Markerview version 1.2.1. (Sciex)) facilitated PCA of 60 MRM LC-ESI-MS^2^ HMO data sets derived from analyses of 2 × 30 HM samples (30 samples from 6 weeks and 30 samples from 16 weeks pp). The following parameters were used by the Markerview software to extract peak areas from MRM LC-ESI-MS^2^ raw data: smoothing half width, 9 points; noise percentage, 50.0%; baseline subtraction window, 2.0 min; peak splitting factor, 2 points; retention time tolerance, 15.00 min; minimum peak intensity, 100 cps; minimum peak width, 4 points; minimum signal/noise ratio, 3:1. Prior to performing PCA, MRM LC-MS^2^ peak areas of specific HMOs were first normalized against corresponding peak areas of the internal standard α-arabinopentaose. Then, normalized peak areas were converted into their natural logarithms and finally scaled by mean centering. Subsequently, PCA was performed. To investigate if abundances of individual HMO structures differed significantly between human milk groups as identified by PCA, non-parametric Kruskal-Wallis *H* testing [77] was performed with SPSS statistics software version 19.0.0 (IBM, Armonk, USA). Non-parametric Kruskal-Wallis *H* testing was applied as prior exploration of data sets by the Shapiro-Wilk test [78] indicated non-normal distribution of HMOs. The null hypothesis for non-parametric Kruskal-Wallis *H* testing was that medians of detected responses *R* for particular HMOs were the same between the milk groups. The considered significance level *α* was 0.05 and the confidence interval was 95%. The same LC-ESI-MS^2^ HMO raw data sets as for PCA were used, but quality of integration was carefully inspected and manually re-adjusted if peaks were not fully integrated using automatic integration. Welch’s *t* test [79] was applied to investigate if significant differences between HMO peak areas (normalized against the internal α-arabinopentaose) were existing between major HM groups and possible subgroups. Significance was reported if calculated *P* values were < 0.05 (*α* level of 0.05).

Moreover, variations in HMO abundances between HM samples from either 6 or 16 weeks pp were statistically elucidated by using Student’s *t* test [80, 81] and Welch’s *t* test. Prior to *t* testing, all data were interrogated by Levene’s test for homoscedasticity [82]. Peak areas normalized against the internal α-arabinopentaose were used as input for calculations. Significance was stated if calculated *P* values were < 0.05 (*α* level of 0.05). Only HMO structures were considered which exhibited fold changes above 0.1 between the decadic logarithms of arithmetic mean at 6 compared with 16 weeks pp. For HMOs showing significant variations under these conditions, the differences between arithmetic mean values between 6 and 16 weeks pp of individual HMOs were expressed in percentages relative to the 6-week values. Results were displayed as bar charts created with Microsoft Excel for Office 365.

## Results and discussion

Our MRM LC-ESI-MS^2^ approach with enhanced specificity for fucose positional HMO isomers was applied to 60 unknown human milk samples. These milks originated from 30 donors resident of southern Germany which were enrolled in the INFAT study [74]. Specimens were randomly selected from a larger control group of women not subjected to dietary intervention during the study. Per donor, two human milk samples from two different stages of lactation (6 and 16 weeks post-partum) were analyzed.

**Fig. 2:**
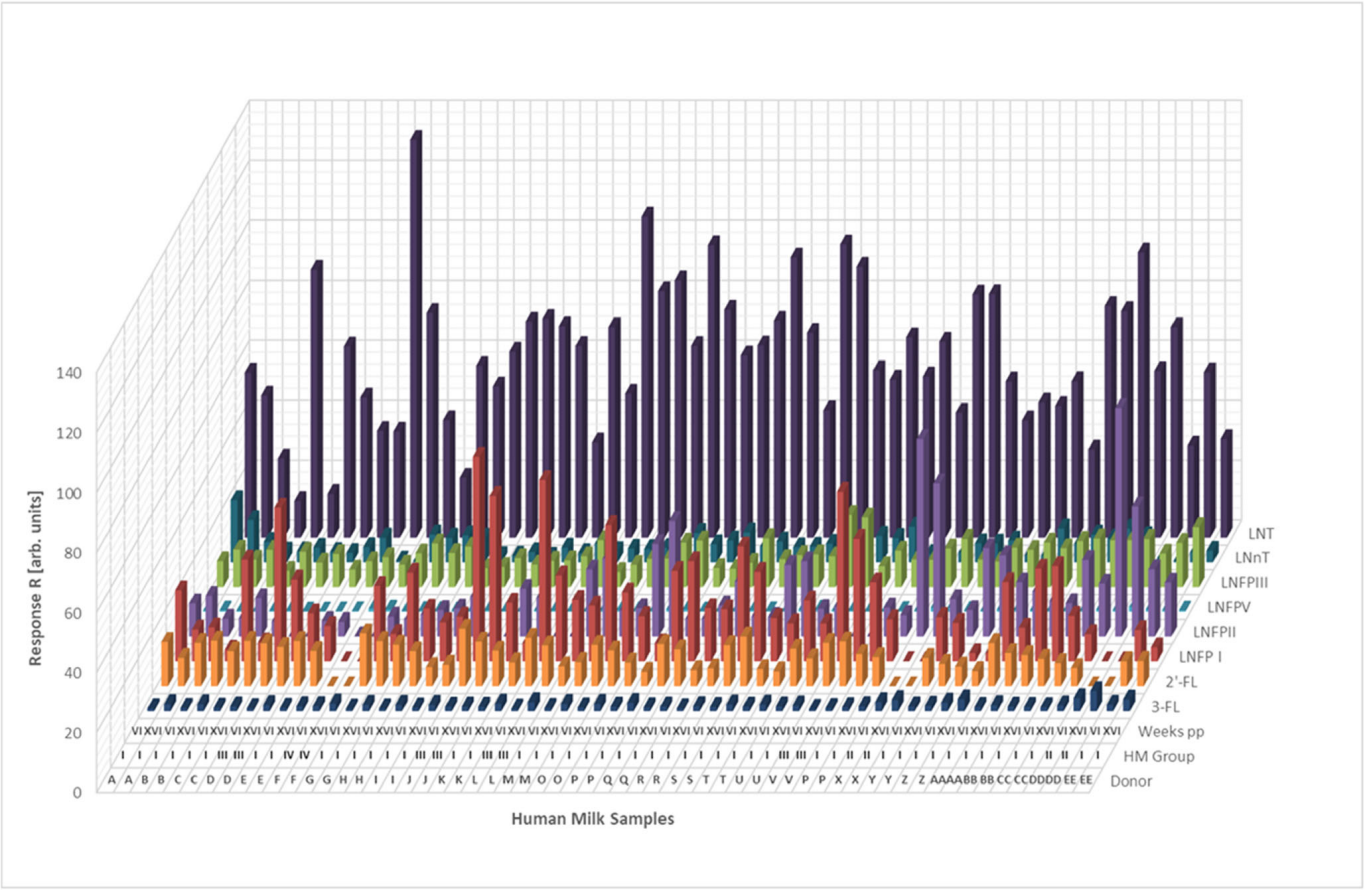
HMOs detected across 60 HM samples with LC-ESI-MS^2^ (*N* = 30 donors, 2 stages of lactation (6 weeks and 16 weeks) per donor). Responses *R* are not strictly equimolar and ranged between 0 and 132. HMO responses below LOD are reported with a zero value. Individual milk donors are indicated by different capital letters A–EE, HM groups by Roman numbers I–IV, and stages of lactation by Roman numbers (VI = 6 weeks pp, XVI = 16 weeks pp)

**Fig. 3:**
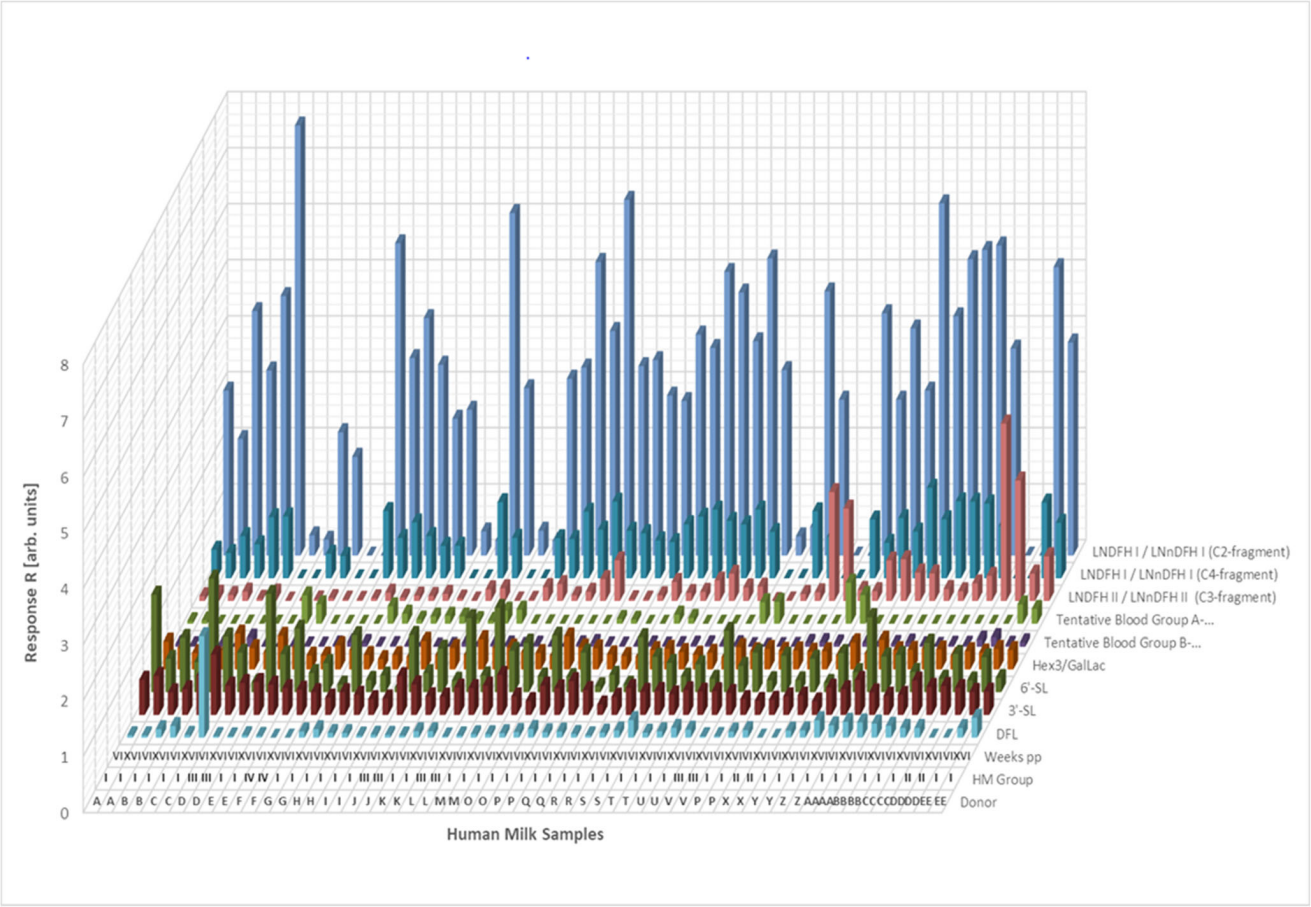
HMOs detected across 60 HM samples (*N* = 30 donors, 2 stages of lactation (6 weeks and 16 weeks) per donor) with MRM LC-ESI-MS^2^. Responses *R* are not strictly equimolar and ranged between 0 and 7.7. HMO responses below LOD are reported with a zero value. Individual milk donors are indicated by different capital letters A–EE, HM groups by Roman numbers I–IV, and stages of lactation by Roman numbers (VI = 6 weeks pp, XVI = 16 weeks pp)

Figure 2 and Figure 3 summarize the resulting MRM LC-ESI-MS2 responses *R* (see “Experimental section”) for HMO structures as detected across the 60 studied HM samples. These major HMO structures cover approximately > 50% of all HMOs contained in HM by quantity. It is noteworthy to mention that the applied semi-quantitative MRM LC-ESI-MS2 approach does not yield equimolar responses for all investigated HMO structures. This is due to many factors including variations in ionization efficiency and fragmentation yields between specific HMOs. Nevertheless, relative differences in HMO concentration between samples or different stages of lactation can be accurately followed for individual HMO structures. Moreover, for some HMO isomers like LNFP I, II, and III, the MRM LC-ESI-MS^2^ responses are similar enough to discriminate between biological abundances of these HMOs. This allows e.g. for reliable milk group assignment [73]. Also other HMOs like 3′SL and 6′-SL show quite similar MRM LC-ESI-MS^2^ responses which enable reasonable direct comparisons of relative abundances. The same is true for LNT and LNnT.

Human milk groups shown in Fig. 2 and Fig. 3 were assigned following the “manual approach” (see “Experimental section”) based on the absence or presence of LNFP I and LNFP II. Hence, 77% of all HM samples (= 23 donors) could be binned into HM group I, 13% (= 4 donors) into HM group III, 7% (= 2 donors) into HM group II, and 3% (= 1 donor) into HM group IV. These proportions did not change when the PCA-based milk grouping approach was applied alternatively (see below).

### Principal component analyses of HMO MRM LC-ESI-MS^*2*^ data

As outlined in the “Experimental section”, yielded LC-MS data were also subjected to unsupervised multivariate statistics using principal component analysis (PCA). This was done in an attempt to investigate three aspects:

1. the distinction of HM samples into (sub)groups not discovered by the manual approach,
2. the effect of progressing lactation on human milk group proportions between 6 and 16 weeks pp, and
3. the variations of Le/Se status–related or Le/Se status–independent HMOs between HM groups.

### PCA-based grouping of HM milk samples

**Fig. 4:**
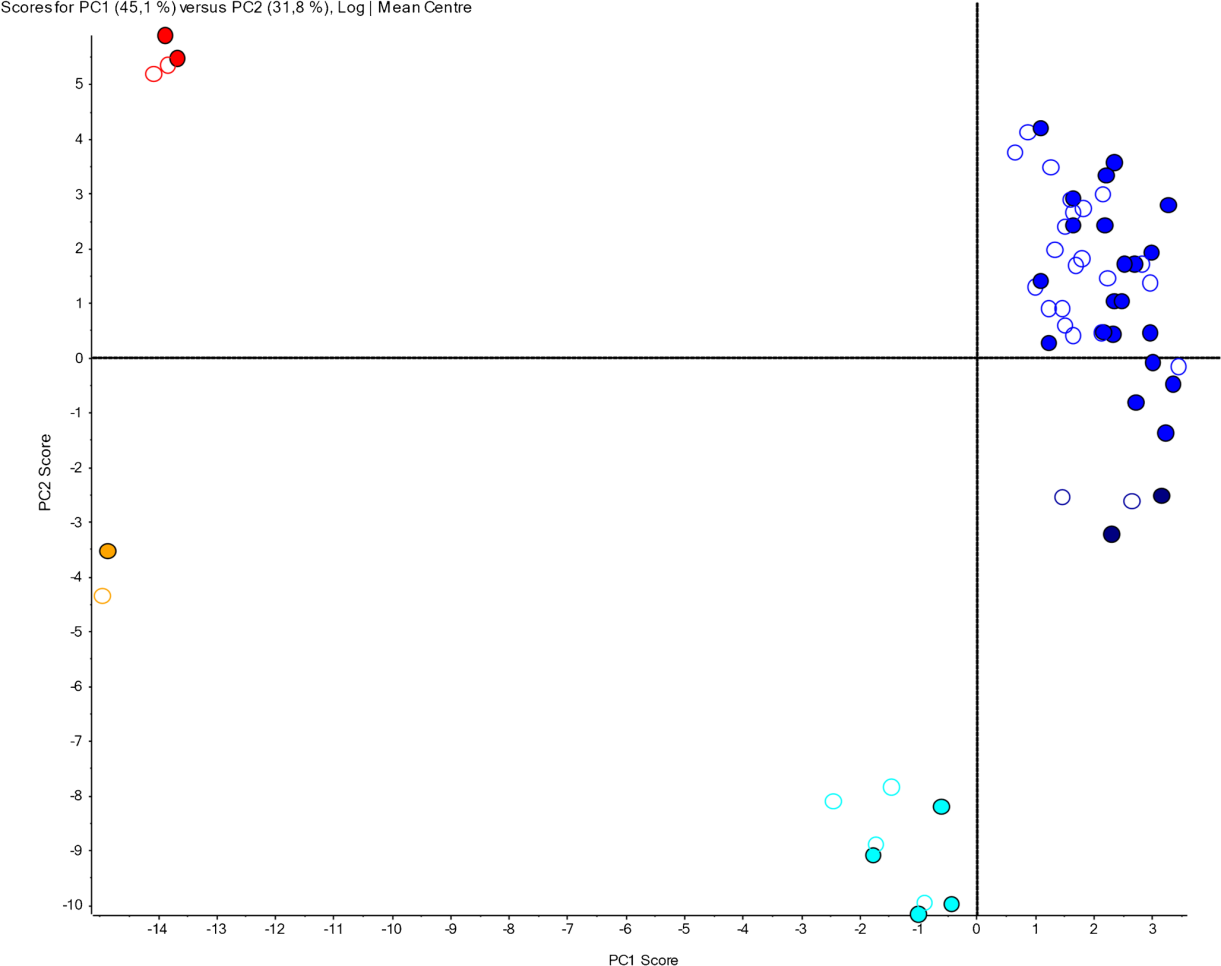
PCA evaluation of MRM LC-ESI-MS^2^ data from 2 × 30 human milk samples (collected at 6 and 16 weeks pp). Every HM sample is represented by a dot in the PCA scoring plot depicted below. Blue and dark blue circles, cluster 1/group I human milks; red circles, cluster 2/group II human milks; cyan blue circles, cluster 3/group III human milks; and yellow circles, cluster 4/group IV human milks. Filled circles represent HM samples collected at 6 weeks pp. Open circles represent HM samples collected at 16 weeks pp

Figure 4 shows the resulting PCA score plot which could explain approximately 77% of variances in the MRM LC-ESI-MS^2^ data set (PC 1, ~ 45%; PC 2, ~32%). Notably, four separate sample clusters distinguished from each other (blue, red, cyan, and orange dots). After post hoc inspection of LC-MS data sets for Se/Le gene–dependent milk group marker HMOs like 2′-FL, LNFP I, LNFP II, DFL, LNDH I, and LNDH II, each cluster could be unambiguously assigned to one of the 4 commonly known human milk groups (HM). Blue and dark blue circles in Fig. 4 represent samples belonging to HM group I, red circles HM group II, cyan circles milk group II, and orange circles milk group IV. Filled circles are HM samples collected at 6 weeks after delivery whereas open circles represent HM samples from 16 weeks pp.

Unexpectedly, within cluster I (blue circles, HM group I), a tendency towards formation of a possible subgroup (full and open dark blue circles in Fig. 4) became visible. We named this subgroup “HM group Ia.” To investigate if responses of certain HMOs might differ significantly between HM group I and HM group Ia, we performed *t* testing as described in the “Experimental section.” Thereby, the following findings were obtained: At 6 weeks post-partum, LNFP V/VI, LNFP II, LNDFH II or LNnDFH II (C3-fragment), LNFP III, and 2′-FL varied significantly (all *P* < 0.001), and also 3′-SL (*P* = 0.03) and DFL (*P* = 0.04) were significantly different in abundance. At 16 weeks pp, LNFP V/VI, LNFP II, 3-FL, LNDFH II, or LNnDFH II (C3-fragment) exhibited significant abundance changes (all *P* < 0.001). Also levels of tentative blood group B-tetrasaccharide/(Hex)3(Fuc)1 (*P* = 0.002) and 2′-FL (*P* = 0.003) were found to be significantly different. In general, median values for Le+/FUT 3-related HMOs like LNFP II and LNDFH II were always lower in HM group Ia compared with HM group I. On the other hand, Se+-dependent HMOs like 2′-FL appeared in much higher levels in HM group Ia compared with HM group I.

The observed HM group Ia subset is concordant with similar findings from Dutch and Chinese human breast milks [54]. In 2018, Elwakiel et al. characterized HMO patterns derived from 30 HM from different Chinese donors via CE-LIF. Milk from each donor was sampled between the first and 20th week after birth. For comparison, also 28 different Dutch breast milks which had been donated approximately at 4 weeks pp to a milk bank were analyzed. Hierarchical clustering of Chinese milk HMO patterns revealed a division of HM group I into two subgroups. These subgroups differed significantly in concentrations of 2′-FL, DFL, LNFP I, and LNFO. However, separation of Dutch Se+Le+ breast milks into subdivisions was driven by 2′-FL, LNT, and LNFO. Unlike in our study, 3-FL could not be evaluated as it was removed during sample pre-treatment. Also, no information was provided about the impact of LNFP II, LNFP III, LNFP V, or LNDFH II.

In sum, the “manual” approach for assignment of HM groups and the unsupervised PCA assignment resulted in the same 4 major milk groups with the same relative proportions of samples and also identical individual samples assigned to these major groups.

**Fig. 5:**
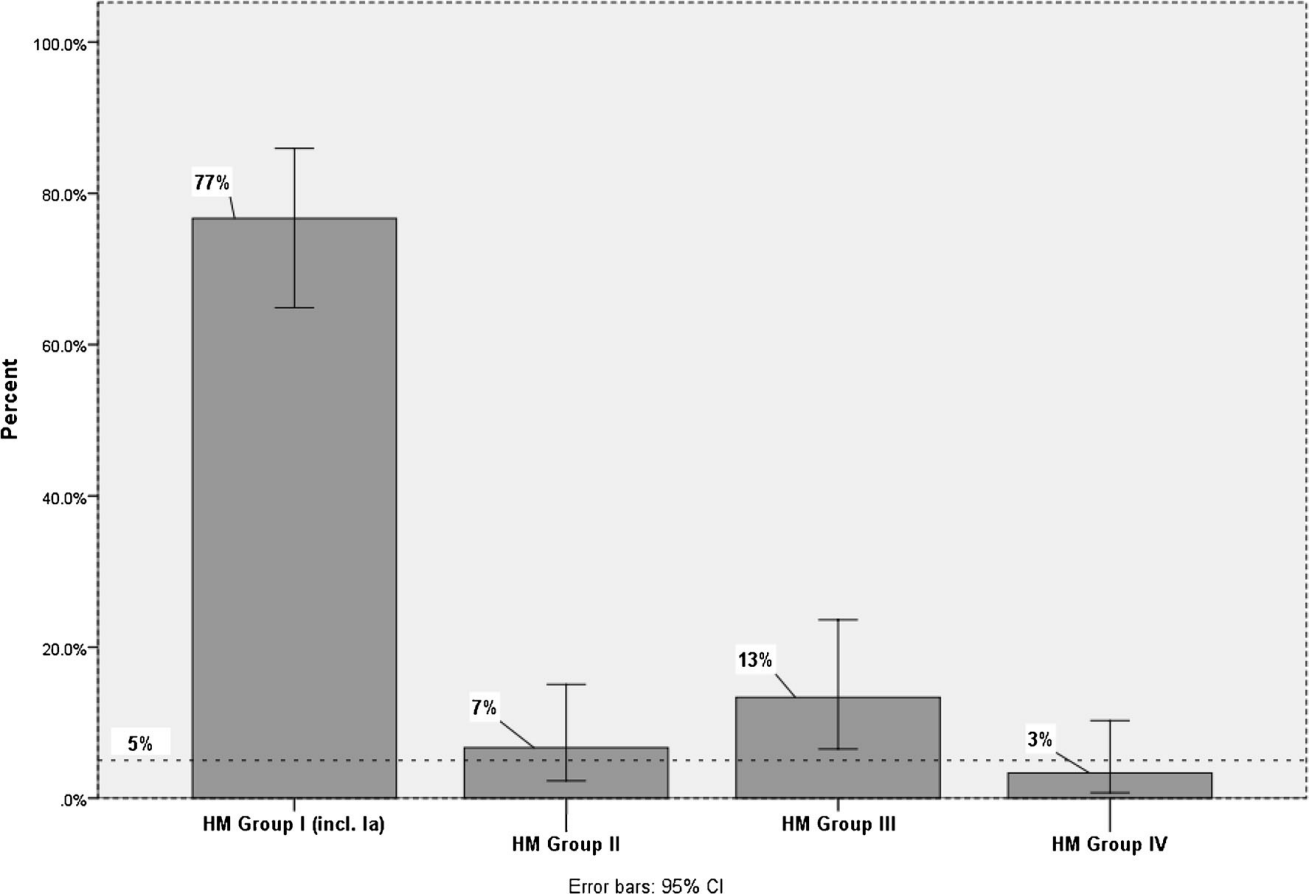
HM group distribution in percentages based on MRM ESI-LCMS^2^ analysis of 60 HM samples. Error bars represent 95% confidence intervals

Figure 5 summarizes the resulting proportions of the 4 milk groups in percentages after characterization of HM samples by MRM LC-ESI-MS^2^. HM group Ia (contributing 5% to the total HM group I +Ia) was not considered separately but accounted for in one HM group labeled as HM group I + Ia. This was done to be consistent with the number of milk groups published earlier [52] and to acknowledge the preliminary nature of HM group Ia identification: In our approach, this HM subgroup consisted of only 4 HM samples from 2 donors. Thus, future confirmation by analysis of much more HM samples is warranted. The final PCA-based proportions of human milk groups (Fig. 5) were 77% (HM group I + Ia), 7% (HM group II), 13% (HM group III), and 3% (HM group IV). This result was identical to the proportions yielded by “manual” assignment relying on the presence or absence of LNFP I and LNFP II (see above). A comparable frequency of ~ 76% for human milk group I was reported by Tonon et al. in 2019 [83] for mature Brazilian human milks from 78 women. Noteworthily, the proportions for group II and group III of ~ 12% each differed from our results especially with regard to group II. This may be explained by regional differences or varying ethnicities between Germany and Brazil. Furthermore, in 2019, Samuel et al. [84] reported new findings for HM group distributions for milks collected during the first 4 months of lactation from 290 subjects across Europe (not including Germany). In their publication, 72% of donors could be assigned to HM group I, whereas ~ 18% of donors belonged to HM group II, ~ 7% to HM group 3, and ~ 4% to HM group IV. Again, differences between our findings and these numbers may be attributed to regional, ethnic, or yet unknown genetic or exogenous factors.

Similar proportions for human milk groups as found by us were also received by analyses of maternal Lewis blood group determinants directly in plasma of French (Oriol et al. 1986 [39]) or German (Thurl et al. in 2010 [16]) donors. Oriol reported values of 70% for Le (a-b+c-d-) phenotypes, 9% for Le (a-b-c-d+), and 1% for Le (a-b-c+d-). Thurl et al. tested blood specimen from 30 subjects but only for Le a and Le b epitopes and found the following distribution: 73% Le (a- b+), 17% Le (a+ b-), and 10% Le (a- b-). In general, the outcome of our study for HMOs and the results of Oriol et al. and Thurl et al. re-confirm the relationship of the Le blood group system and the maternal Le/Se status–dependent HM group system (see also Table 1). In this context, HM group II seems to be underrepresented in our data as only 7% of samples were assigned to this group. The corresponding Lewis blood group proportions published by Oriol et al. and Thurl et al. were 20% (Le a+b-c-d-) and 17% Le (a+b-), respectively. This difference may be attributed to the different geographies from which HM samples had been derived (Germany vs. France) or to different proportions of ethnicities in the probed sample sets. Also, variations introduced by the targeted biomolecules themselves (human milk oligosaccharides (this study) vs. erythrocytes) might be reasons for deviating findings. The set of fucosyltransferases (FUTs) affecting formation of Le epitopes on erythrocytes is related but not identical to the FUTs active in HM. Moreover, the relatively low number of 30 donors in our study might have contributed to the observed variations from results found by Oriol et al. in 1986, too. Therefore, in future, the parallel analysis of statistically meaningful numbers of blood and HM specimen from donors of the same geographies and ethnicities may be helpful to mount further evidence.

### PCA-based investigation of effects related to progression of lactation

In the PCA score plot depicted in Fig. 4, only minor shifts in ordination of HM samples could be observed between sampling at either 6 (filled circles) or 16 weeks (open circles) after birth. On the contrary, the pronounced distances between HM group clusters I–IV did persist despite changes of lactational stages. Also the assignment of individual HM samples to HM groups I–IV stayed the same at 6 and 16 weeks pp. Consequently, no percentual change in milk group ratios could be attributed to progression of lactation. Thus, we conclude that at least between 6 and 16 weeks pp, progression of lactation has no influence on milk group distribution and milk group–related diversity (but not abundance) of particular HMOs.

### Variation of Le/Se status–related or Le/Se status–independent HMOs between milk groups

**Fig. 6:**
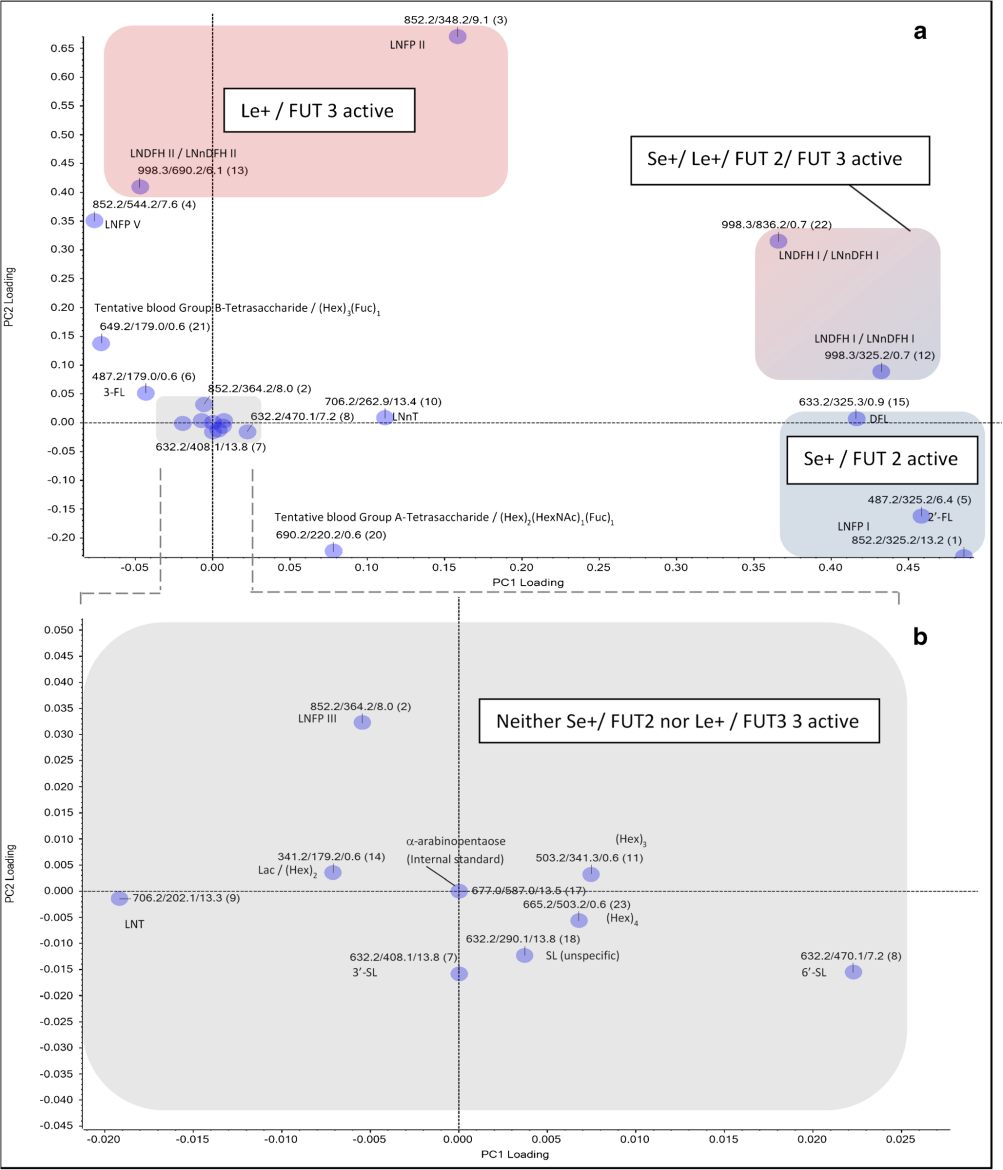
**a** PCA loading plot based on LC-ESI-MS^2^ responses *R* for 21 MRM transitions of individual, partly isomeric HMOs as detected in 2 × 30 human milk samples collected at 6 and 16 weeks pp. HMO-specific MRM transitions are represented by individual dots including information about precursor masses, fragment masses, and retention times. The order number of transition in MRM scan is reported in parentheses. **b** Magnification of gray shaded section in (**a**) depicting MRM transitions of HMOs with no or low contribution to clustering of HM

The PCA loading plot displayed in Fig. 6 helped to reveal specific HMO compounds which were driving the separation into 4 major milk group clusters most: The highest impact on separation of milk groups was exerted by a set of MRM LC-MS^2^ transitions specific for LNFP I, 2′-FL, and DFL (light blue rectangle). These HMOs are linked with a secretor-positive (Se+) genetic maternal status. To a lower degree, also LNDFH I/LNnDFH I transitions (light red and blue rectangle), which are concatenated with a positive secretor and Lewis gene status (Se+/Le+), had a strong impact on HM grouping. HM group separation was less but still considerably influenced by MRM transitions of Le-positive (Le+) status–related HMOs like LNFP II and LNDFH II/LNnDFH II (light red rectangle). Intriguingly, another set of Se and Le gene–independent HMOs like LNFP V/VI, and LNnT, and also the tentative blood group A and B tetrasaccharides seemed to contribute to clustering as well, but to an even lesser extent than the Le/Se gene–linked glycans. Other compounds like LNFP III, lactose/(Hex)_2_, (Hex)_3_, and 6′-SL have almost no effect on group separation. This means that the latter structures are quite stable in presence and abundance across all probed HM samples. Variability of Le and Se gene–regulated HMOs is much higher.

The observations explained above were further substantiated by non-parametric statistics. The null hypothesis that distribution and abundance of particular HMOs are the same across the 4 human milk groups as distinguished by PCA was rejected for all measured Le/Se-dependent but also some Le/Se-independent HMOs by non-parametric statistics (Kruskal-Wallis method, see “Experimental section”) as well. As a result, actually 12 HMO structures differed significantly in abundance between human milk groups I–IV (see Table 2). Moreover, some HMOs could only be detected in specific milk groups (see column 3 in Table 2 or Fig. 2 and Fig. 3). If HMOs were detectable in more than one milk group, the group with the highest median was highlighted by red bold letters in Table 2. For example, 2′-FL and LNFP I could both be detected in HM groups I and III but medians were higher in HM group III. Thus, Roman number III is displayed in bold red for both structures.

**Table 2:**
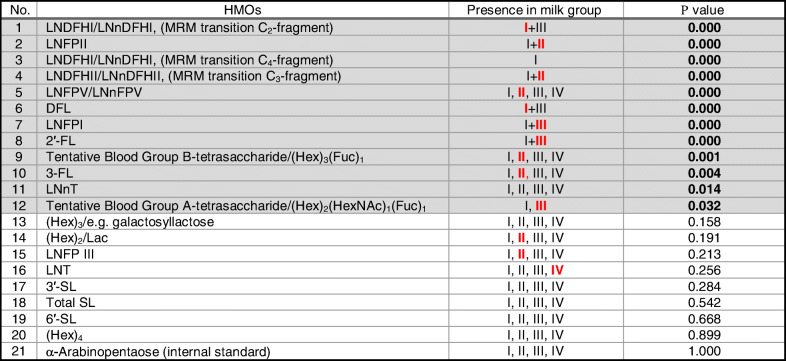
Milk group–specific presence of individual HMOs. *P* values < *α* indicate statistically significant differences between milk groups I, II, III, and IV found for median values of 12 particular HMOs (nos. 1–12, gray shaded). *α* = 0.05, non-parametric Kruskal-Wallis test, *N* = 2 × 30 samples (from 6 and 16 weeks pp). Bold red letters in column 3 indicate elevated median values of specific HMOs in this particular milk group relative to other milk groups

Noteworthily, HMOs related to a positive maternal genetic Se status (Se+), positive Le status (Le+), or positive Se and Le status (Se+Le+) such as 2′-FL, DFL, LNFPI, LNFP II, LN(n)DFH I, and LN(n)DFH II exhibited significantly different levels between milk groups (significance level *α* = 0.05, see Table 2). The latter findings approximately corroborate data published e.g. by Thurl et al. in 2010 [16] for milk group–dependent abundances of distinct neutral, fucosylated HMOs.

This outcome complies with the well-accepted biological expectation that distribution of specific HMO structures should differ in HM samples due to known differences in maternal Le/Se status which can occur between individual milk donors: If the maternal Secretor and/or Lewis gene is active, it will encode expression of fucosyltransferases FUT 2 and/or FUT 3 and subsequent synthesis of either α1,2- and/or α1,4-fucosylated HMOs in the mammary gland.

As a result, the presence or absence of α1,2- and/or α1,4-fucosylated human milk OS should lead to formation of 4 different milk groups defined by presence (or absence) of α1,2- and/or α1,4-fucosylated HMOs. As mentioned before, not only the expected appearance of these different milk groups but also significant variations in α1,2- and α1,4-fucosylated oligosaccharides between the 4 HM groups could be confirmed after interrogation of the LC-MS results through PCA and non-parametric statistics (see Table 2). More surprisingly and in addition, PCA and non-parametric statistical testing suggested that some HMOs such as α1,3-fucosylated 3-FL and LN(n)FP V or e.g. non-fucosylated LNT which are all independent from maternal Le/Se status did also significantly vary between HM groups.

A further examination of milk groups for specific HMO structures revealed another unexpected finding: the appearance of the MRM signal for LNDFH I based on its C_2_ fragment (see Table 3) in milk group III samples (with lower abundance than in milk group I). This finding is in contradiction with the published knowledge describing a lack or strongly reduced activity of FUT 3 for Le-negative HM group III donors. FUT III would be needed in biosynthesis to append a fucose in α1,4 glycosidic linkage to the GlcNAc in the type I LNT backbone of LNDFH I. An explanation for this paradox may be provided by the fact that the used LNDFH I C_2_-fragment-based MRM transition obviously picks up both the backbone type I and type II isomers of LNDFH I. Therefore, the LNDFH I variant determined in the group III milks is most probably LNneoDFH I, also named LNDFH III. LNDFH III consists of a type II LNnT backbone and the α1,4-linked fucose present in LNDFH I is replaced by a α1,3-linked fucose. The latter structure is FUT 3 independent and could therefore be present also in HM group III samples. In contrast, LNDFH I (with type I backbone) is detected only in group I milks as expected if the MRM transition of the LNDFH C_4_-fragment is monitored. Thus, the latter transition is obviously more specific and suitable for correct identification of LNDFH I.

**Table 3:**
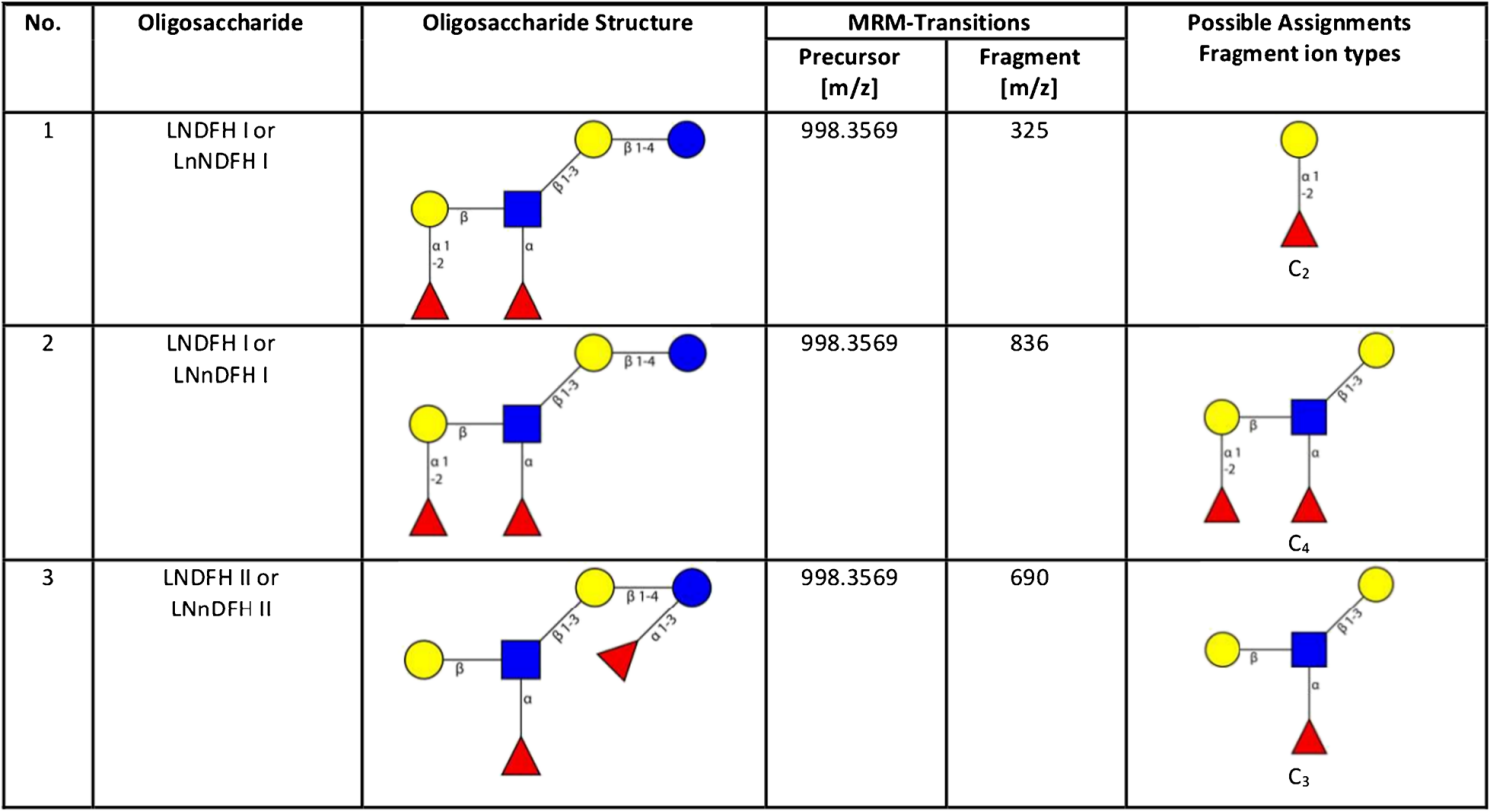
Isomeric LNDFH structures, possible tandem MS fragment ion types, and related MRM transitions as utilized in the applied HMO LC-ESI-MS^2^ method

### Relative HMO abundance changes between 2 stages of lactation (6 and 16 weeks post-partum)

Another important aspect which may contribute to functional adaptation of human milk during staging of lactation is the change of concentrations observed for particular HMOs. To further investigate such staging effects, we compared the human milks which were either collected at 6 or 16 weeks after parturition. This was done with no distinction between milk groups.

**Fig. 7:**
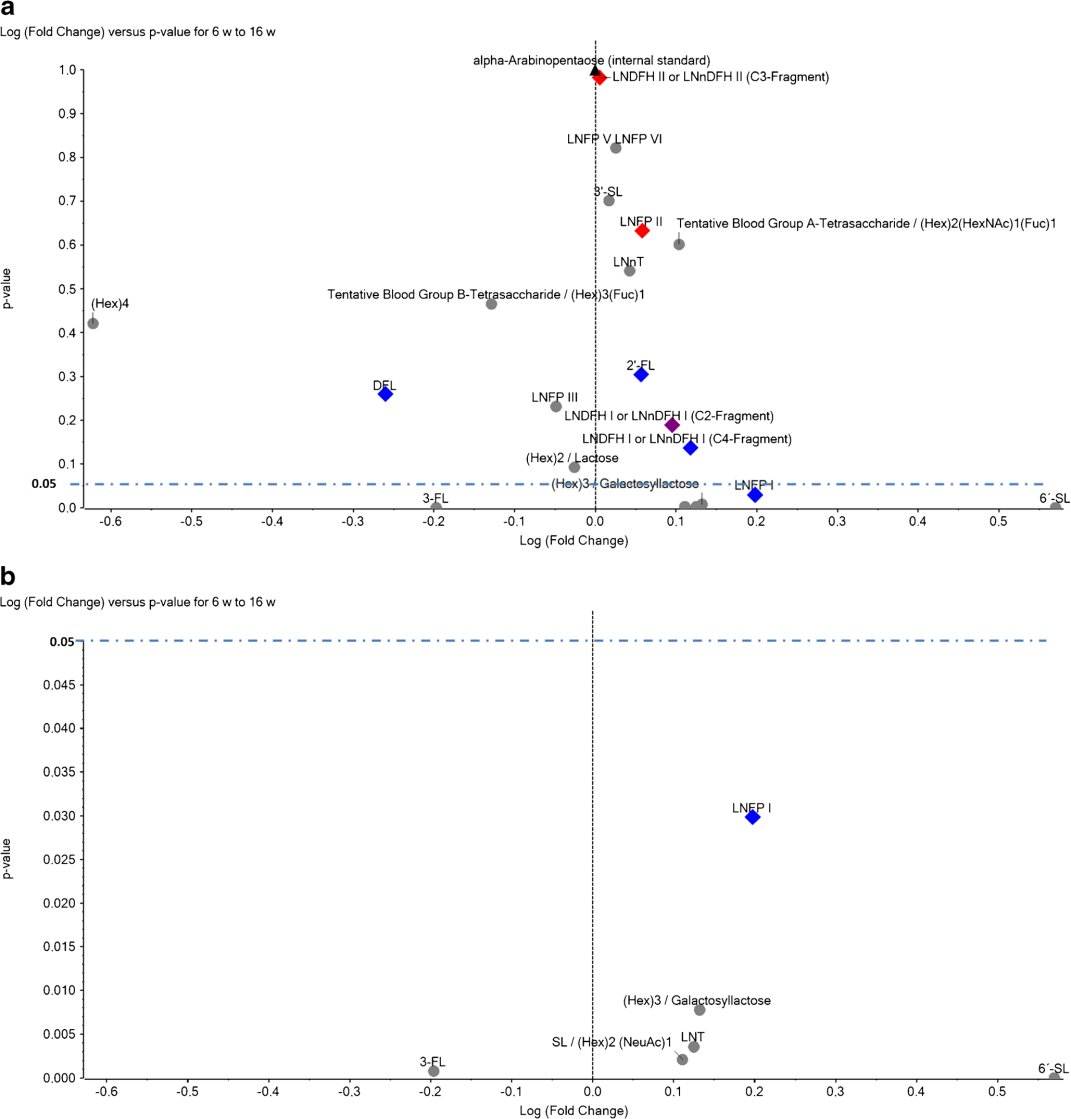
Variations of individual HMO levels between stages of lactation. **a** *P* values for variations in HMO mean values between 6 and 16 weeks pp were plotted over respective fold changes. Negative values for fold changes indicate an increase of HMO levels over time, and positive values decrease. **b** Section of (**a**) displaying only significantly changing HMOs (*P* < *α*). Red diamonds, Le+-dependent HMOs; blue diamonds, Se+-dependent HMOs; purple diamonds, Se+- and Le+-dependent HMOs; gray dots, Le/Se-independent HMOs; and black triangle, internal standard alpha-arabinopentaose

As shown in Fig. 7a and b, only six HMO structures were changing significantly in abundance between the lactational stages at 6 and 16 weeks post-partum (*α* level of 0.05; fold change threshold of ± 0.1). This finding could be confirmed for 6′-SL, 3-FL, LNFP I, and (Hex)_3_ also with Wilcoxon signed-rank test for non-normally distributed, interdependent samples (data not shown). Among these significantly changing HMOs, only one glycan (LNFP I) was related to maternal Se+ status. LNFP I decreased in its relative abundance. Additional HMO compounds including 6′-SL, total SL, (Hex)_3_, and LNT showed the same tendency and decreased. In opposition to this trend, 3-FL exhibited an increase in relative abundance between 6 and 16 weeks pp. Except for LNFPI, all the other 5 HMOs were independent from maternal Le or Se status.

**Fig. 8:**
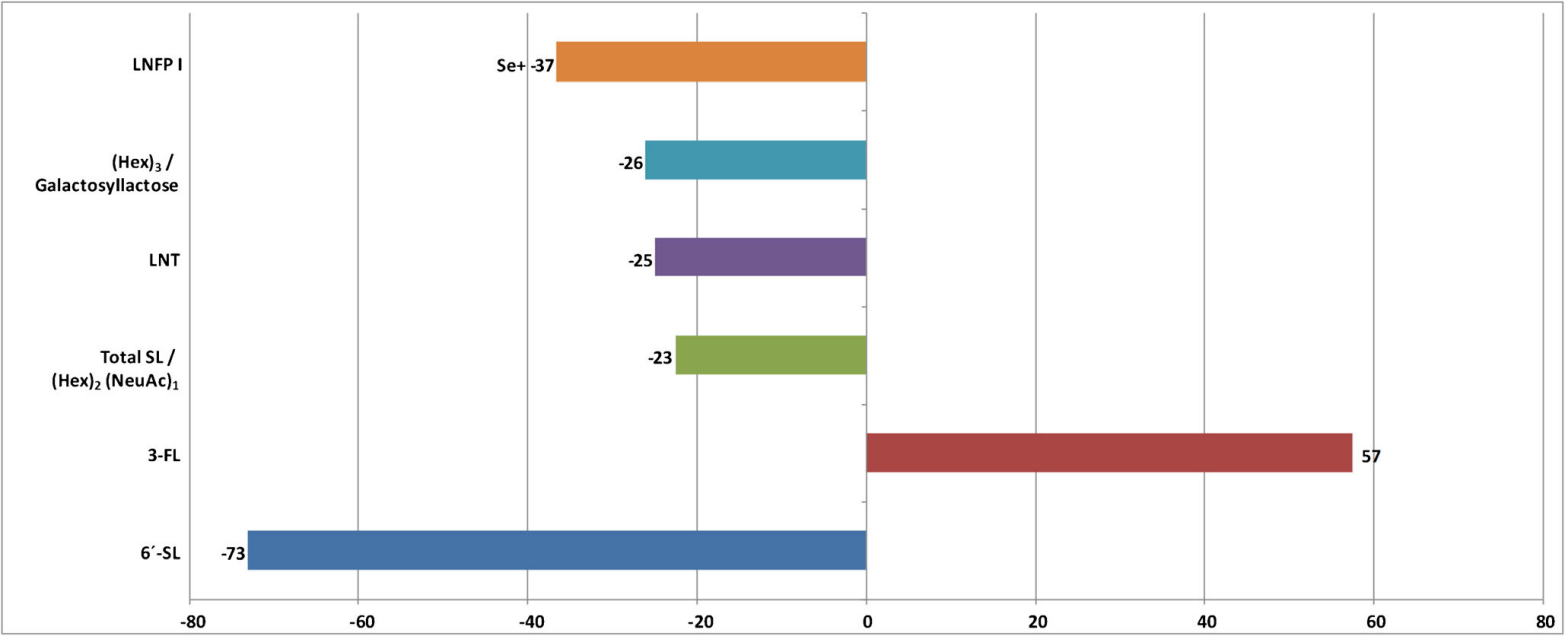
Quantitative differences of HMO mean values with significant level changes (*t* test, *P* < 0.05) between 6 and 16 weeks pp. Level changes are expressed in percentages relative to the mean value at 6 weeks pp. Highest increase over time is found for 3-FL (57%), highest decrease for 6′-SL (− 73%), followed by LNFP I (− 37%)

Figure 8 visualizes quantitative abundance changes for the six selected HMO structures visible in Fig. 7b. Level changes were expressed in percentages relative to the original abundances measured at 6 weeks pp. Here, 6′-SL showed the strongest quantitative variation among all investigated HMOs with a decrease of 73%. On the contrary, 3′-FL increased by approximately 57% during the same time span. Interestingly, in 2016, Seppo et al. [85] published similar data with highly significant shifts of 6′-SL (decrease) and 3-FL (increase) between 0 and 6 months pp. They also showed a negative correlation between LNFP I concentration and increasing duration of breastfeeding. In contrast to our analytical LC-MS-based approach, these authors relied on HPLC analysis of HMOs after labelling with 2-aminobenzamide. Also other reports state a steady increase for 3′-FL levels either between approximately 0 and 8 months pp for HM collected from Chinese women [86] or (at least) between 0 and 4 months pp for HM from Pan-European populations [84]. Furthermore, Austin et al. could show that 2′-FL, 3′-SL, 6′-SL, LNT, LNnT, LNFP-I, and LNFP-V decreased by trend over lactational stages. Although we analyzed human milks from a different geography (Germany), these trends were reflected in our data, too. Another publication by Coppa et al. [18] was in line with our results as well. In the latter study, 3′-SL decreased between 0 and 3 months pp in HM, but the differences in 3′-SL were not significant. Also the 6′-SL concentration declined over the same time range, but in this case and similar to our findings, the concentration changes were significant.

## Conclusions

In this study, application of a novel LC-MRM-MS concept to 2 × 30 mature human milk samples collected at 6 and 16 weeks post-partum was demonstrated. This approach covered (partly isomeric) HMOs up to hexasaccharides and as a novelty also the (tentatively assigned) blood group A and B tetrasaccharides. More than approximately 50% of the HMO quantity present in HM could be characterized.

Principal component analysis (PCA) was successfully utilized for automated interrogation of LC-MS data. The chosen PCA approach allowed for unattended, effortless, and quick assignment of HM samples to one of the 4 currently known human milk groups. The additional advantage of PCA on top of advanced data processing speed and convenience was the immediate visualization and indication for possible HM subgroups. Taking these findings into account, unsupervised PCA seems to be well suited for convenient HM group recognition if applied as described in the “Experimental section”.

Intriguingly, mining of HMO LC-MS data via PCA and comparison with complementary non-parametric statistics revealed first evidence for possible distinction of HM group I into a further HM subgroup termed HM group Ia. Compared with other HM group I samples, HM group Ia specimen had significantly elevated levels of Se+-related HMOs (e.g., 2′-FL) and decreased levels of Le+ structures like LNFP II and LNDFH. Also Le/Se-independent HMOs such as LNFP V/VI or putatively assigned blood group B-tetrasaccharide/(Hex)_3_(Fuc)_1_ were found to differ.

Twelve HMO structures exhibited significantly different abundances between the major 4 HM groups. As expected and in line with contemporary literature, most of those 12 HMOs were related to either a positive maternal genetic Se status, a positive Le status, or a positive Se and Le status. However, also a second set of Le/Se status–independent α1,3-fucosylated compounds like 3-FL and LN(n)FP V or non-fucosylated LNT did vary significantly.

During progression of lactation between 6 and 16 weeks pp, staging effects could be confirmed for 6 distinct HMO structures. Most prominent level changes were revealed for 6′-SL (decrease by 73%) and 3-FL (increase by 57%).

It is challenging to find a rationale why there is a second set of Le/Se gene–independent HMOs, seemingly associated with milk grouping. Neither milk group–relevant active FUT2 or FUT3 genes are required for biosynthetic formation of those human milk glycans. Finally, and in accordance with first interpretation of PCA outcomes, Kruskal-Wallis ANOVA testing could confirm the existence of a third set of human milk oligosaccharides which were assessed to be permanently present across all samples without significant difference between the 4 human milk groups (see nos. 13–21 in Table 2).

Whether milk group–independent, ubiquitously present glycans like lactose/(Hex)_2_, (Hex)_3_, SL, LNT, or LNFP III can therefore be considered as more “essential” with regard to healthy infant development rather than the Le/Se milk group–dependent HMOs is yet still open. At least growing published evidence states their association with relevant early life health benefits like bifidogenic effect, anti-infective efficacy (e.g., directed against parasitic protozoans), and modulation of the developing immune system including less incidence of cows’ milk allergy [68, 85, 87–91]. On the other hand, it may be hypothesized that the synthesis and secretion of the Le/Se-dependent HMOs like 2′-FL, DFL, LNFPI [92–96], or LNPF II [97] could also be interpreted as a complementary additional response of evolution to optimize protection of the infant against specific threats like pathogenic bacteria or viruses.

Diversity and abundance of these pathogens however probably did or does still vary by geography due to socioeconomic and environmental circumstances. Therefore, several tailor-made but geographically variable sets of HMOs (i.e., the Le/Se gene–regulated HMOs) may be required to optimally protect the infant with respect to geographically variable, specific early life threats. The profile of a HMO set fitting optimally to the infants needs may also depend on the infants’ own Le/Se status. The latter aspect is important as the Le/Se status also co-defines glyco-epitopes expressed on gut epithelial linings or other inner surfaces [55]. Pathogens can adhere to these epitopes via glycan-specific receptors and subsequently cause infections e.g. of the gastrointestinal tract. An optimal free human milk OS pattern would therefore resemble all glycan epitopes possibly displayed by the host (the infant) to bacteria or viruses. Thereby, effective blocking of glycan receptors of these pathogens could be achieved and adhesion and subsequent infection could be avoided.

However, if pathogenic threats throughout human evolution exerted a selective pressure on HMO profiles, the evolutional adaption should have manifested itself in geographically different Le/Se gene–dependent HMO patterns. These patterns may still be traceable today in diverse geographic regions or ethnicities. In this sense, some publications propose a possible link between history of human FUT 2 polymorphisms [98] or blood group variations with partly pathogen-driven selective pressures [99]. Moreover, there is at least a growing body of evidence published by e.g. Erney et al. [23] and other authors [100–103] that prevalence of the Se gene and related HMOs like 2′-FL differs in a geographically specific manner.

Whether the idea of “essential” HMOs which are always present across all human milk groups and other milk group–dependent “specialized” e.g. anti-infective HMOs can be substantiated by further studies remains to be elusive and an exciting question. The same applies for the influence of HMO abundances on infants’ healthy development which is significantly changing during lactational staging. In this publication, we investigated only the segment of human milk lactation between 6 and 16 weeks pp. An extended milk sampling including specimen from the first week until 6 or even 12 months pp and a much higher number of samples could reveal more insights in future.

Other biofluids than HM such as fecal slurries or plasma could probably also become amenable to sensitive MRM-LC-MS analyses by slight adaptions of the current sample pre-treatment procedure. For example, additional microfiltration steps or SPE could be exploited in addition or as an alternative to 3-kDA ultrafiltration. Thus in future, more holistic insights may be elicited by improved analyses with regard to systemic effects of HMOs on healthy infants’ development during the relevant first 1000 days of life.

Finally, we hope that the described multiple reaction monitoring (MRM) LC-ESI-MS^2^ methodology might help to study HMO profiles of even larger human milk sample sets e.g. provided by prospective human milk cohorts. If combined with PCA, this in turn may contribute to reveal new associations between variations in HM glycan composition and their impact on healthy infant development and early life immunomodulation.

## Electronic supplementary material

ESM 1 (PDF 647 kb).
