# Supplementary material for: Targeted LC-ESI-MS2 characterization of human milk oligosaccharide diversity at 6 to 16 weeks post-partum reveals clear staging effects and distinctive milk groups
Source: Anal Bioanal Chem. 2020 Aug 14;412(25):6887–907. doi: 10.1007/s00216-020-02819-x (PMC7496073; doi:10.1007/s00216-020-02819-x)
Supplement: Supplementary file 1 — (PDF 647 kb). [file 216_2020_2819_MOESM1_ESM.pdf]

Analytical and Bioanalytical Chemistry

Electronic Supplementary Material

Targeted LC-ESI-MS^2-characterization of human milk oligosaccharide diversity at 6 to 16 weeks post partum reveals clear staging effects and distinctive milk groups

Marko Mank, Hans Hauner, Albert J.R. Heck, Bernd Stahl

Table S1 LC-Gradient of the applied MRM LC-ESI-MS^2 method

Time [min]	A (%)	B (%)
0.00	98.0	2.0
1.00	98.0	2.0
1.54	98.0	2.0
2.64	91.5	8.5
4.18	91.0	9.0
8.00	91.0	9.0
9.50	89.0	11.0
9.82	86.0	14.0
10.70	75.0	25.0
11.80	70.0	30.0
12.90	0.0	100.0
14.22	0.0	100.0
14.66	98.0	2.0
17.74	98.0	2.0

Table S2 Characteristic, isomer-specific MRM transitions for HMOs as applied in our negative ion mode MRM LC-ESI-MS^2 method. Precursor ions are given as monoisotopic m/z values, fragment ions are given as nominal m/z values

**Nr.**	**Oligosaccharide**	**MRM-Transitions**	
		**Precursor [m/z]**	**Fragment [m/z]**
1	2´-FL	487.1668	325
2	3-FL	487.1668	179
3	3´-SL	632.2044	408
4	6´-SL	632.2044	470
5	DFL	633.2248	325
6	LNT	706.2411	202
7	LNnT	706.2411	263
8	LNFP I	852.2990	325
9	LNFP II	852.2990	348
10	LNFP III	852.2990	364

Table S3 Characteristic, composition-specific MRM transitions for oligosaccharides, tentative blood group-related haptens, and the internal standard α-arabinopentaose as applied in our negative ion mode MRM LC-ESI-MS^2 method. Precursor ions are given as monoisotopic m/z values, fragment ions are given as nominal m/z values

Nr.	Oligosaccharide	MRM-Transitions	
		Precursor [m/z]	Fragment [m/z]
11	$(Hex)_2$ / Lactose	341.1089	179
12	$(Hex)_3$ / Galactosyllactose	503.1618	341
13	SL / $(Hex)_2$ $(NeuAc)_1$	632.2044	290
14	$(Hex)_4$	665.2146	503
15	α-Arabinopentaose (internal standard)	677.2148	587
16	Tentative Blood Group B-Tetrasaccharide / $(Hex)_3(Fuc)_1$	649.2197	179
17	Tentative Blood Group A-Tetrasaccharide / $(Hex)_2(HexNAc)_1(Fuc)_1$	690.2462	220
18	LNFP V or LNFP VI	852.2990	544
19	LNDFH I or LnNDFH I	998.3569	325
20	LNDFH I or LNnDFH I	998.3569	836
21	LNDFH II or LNnDFH II	998.3569	690

Fig. S1 Exemplary extracted ion chromatograms yielded after MRM LC-ESI-MS^2 analyses of pure HMOs standards (a) LNFP III, b) LNFP II, c) LNFP I, d) LNT, e) LNnT) and e) HMOs in a group I HM specimen selected from the INFAT study sample set. Traces and assignments of most abundant HMOs: RT window 0-5min: Lactose/Hex2 (brown), 3-FL (pink); RT window 5-8min: DFL (blue), 2'-FL (light blue), 6'-SL (green); RT window 8-12 min: LNFP III (red), LNFP II (green); RT window 12-15 min: LNFP I (black), LNT (turquoise), LNnT (blue), 3'SL (green), internal standard α-Arabinopentose (red)

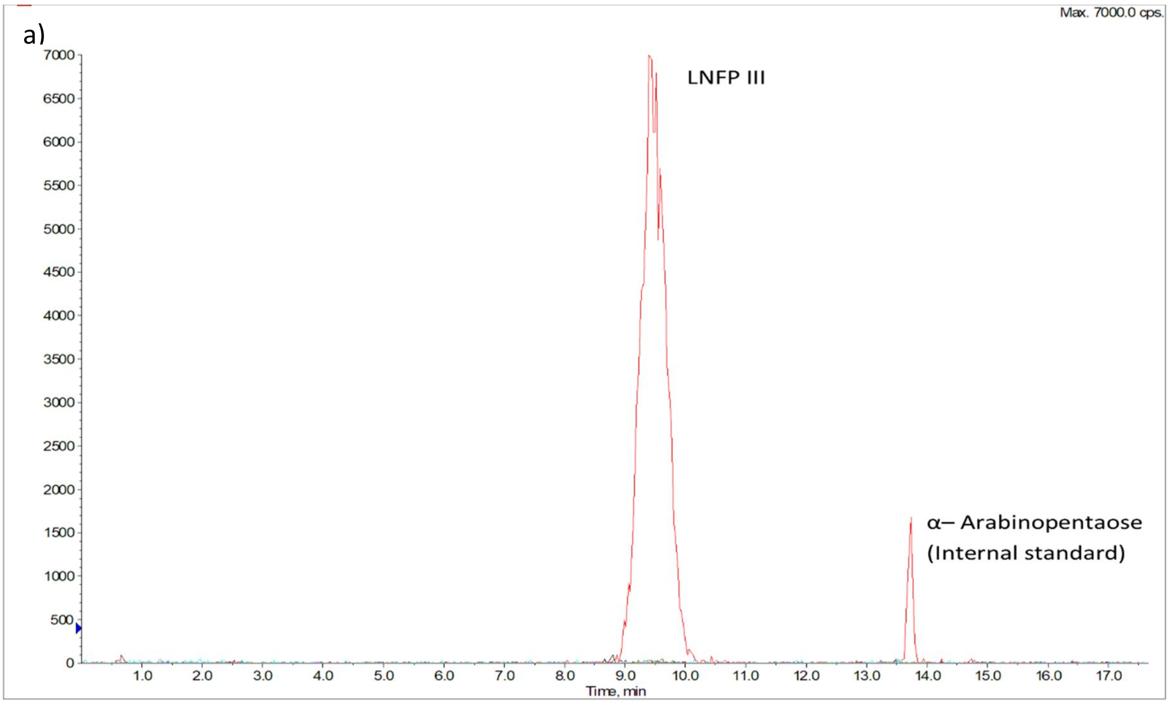

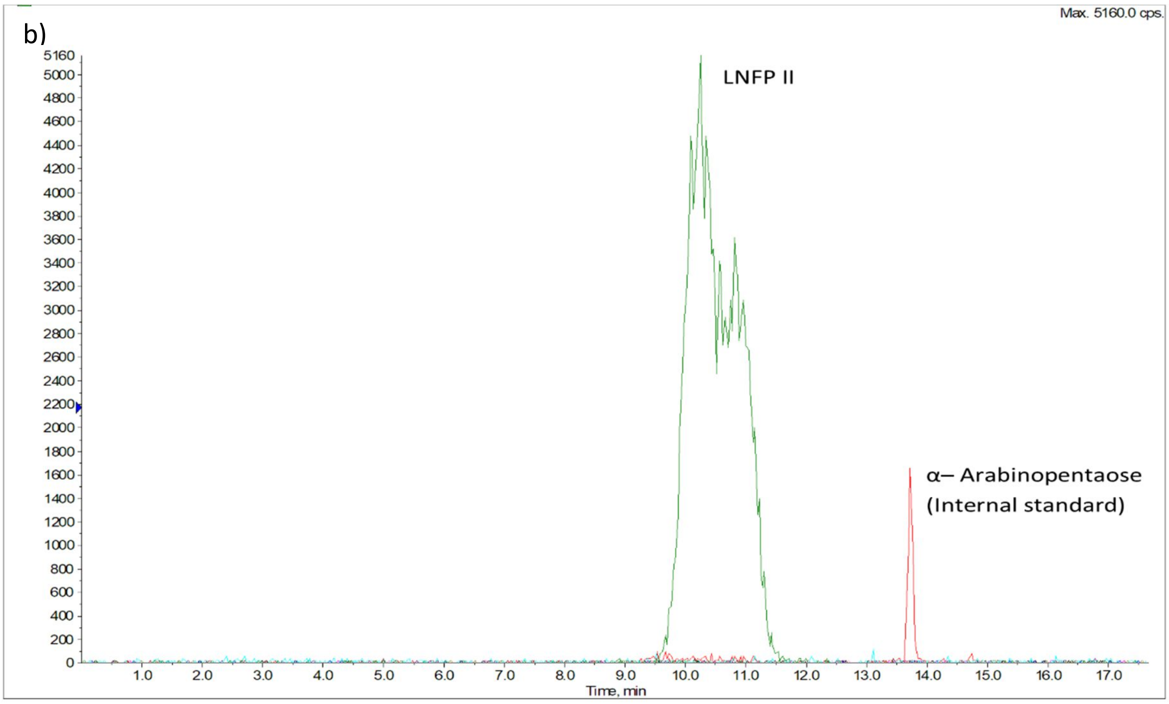

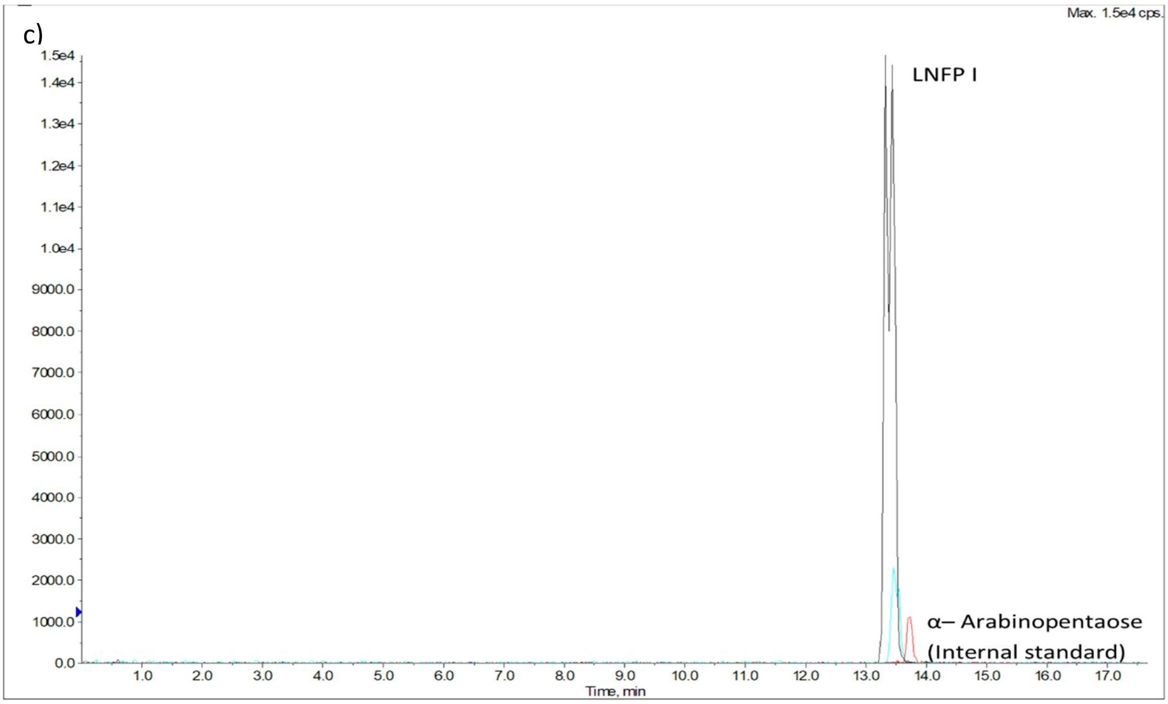
c)
Max. 1.5e4 cps.
LNFP I
α– Arabinopentaose
(Internal standard)
Time, min

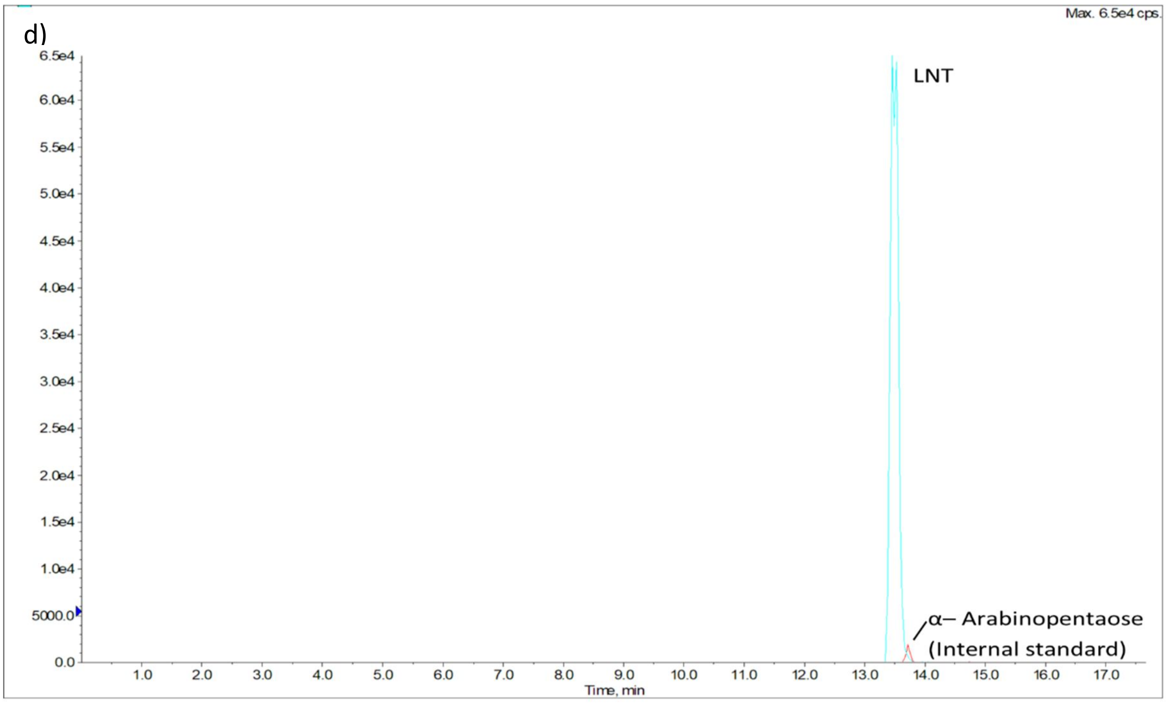
d)
Max. 6.5e4 cps.
LNT
α– Arabinopentaose
(Internal standard)
Time, min

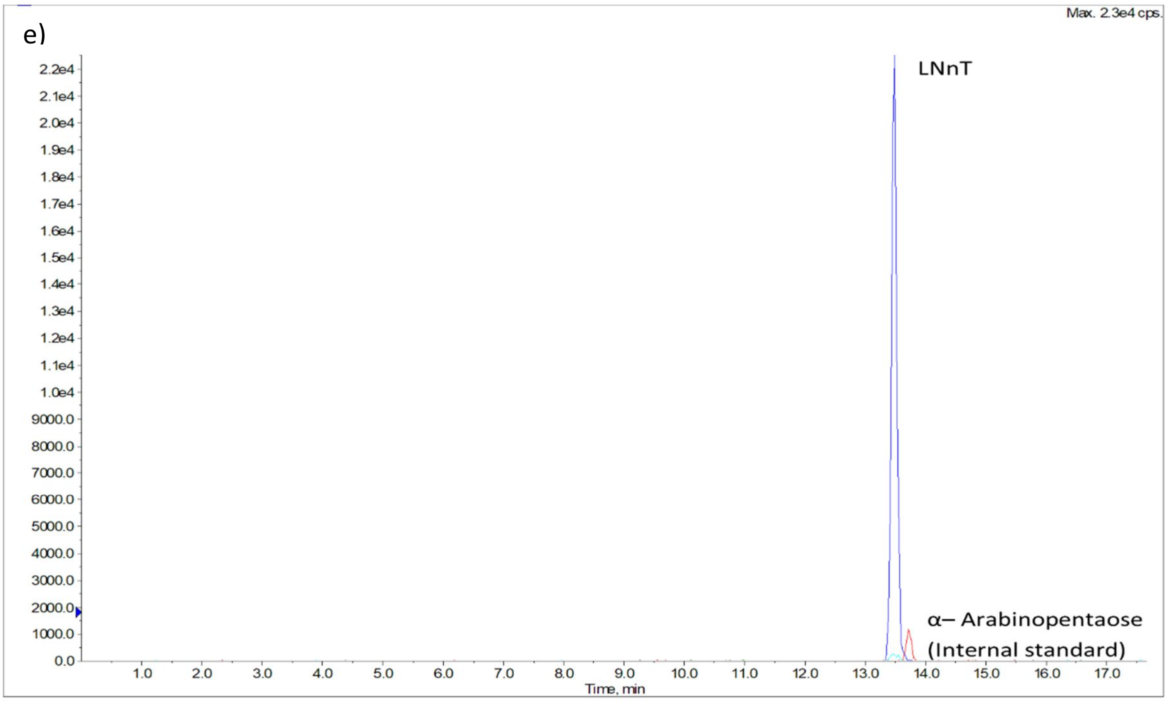
e)
Max. 2.3e4 cps.
LNnT
α– Arabinopentaose
(Internal standard)
Time, min

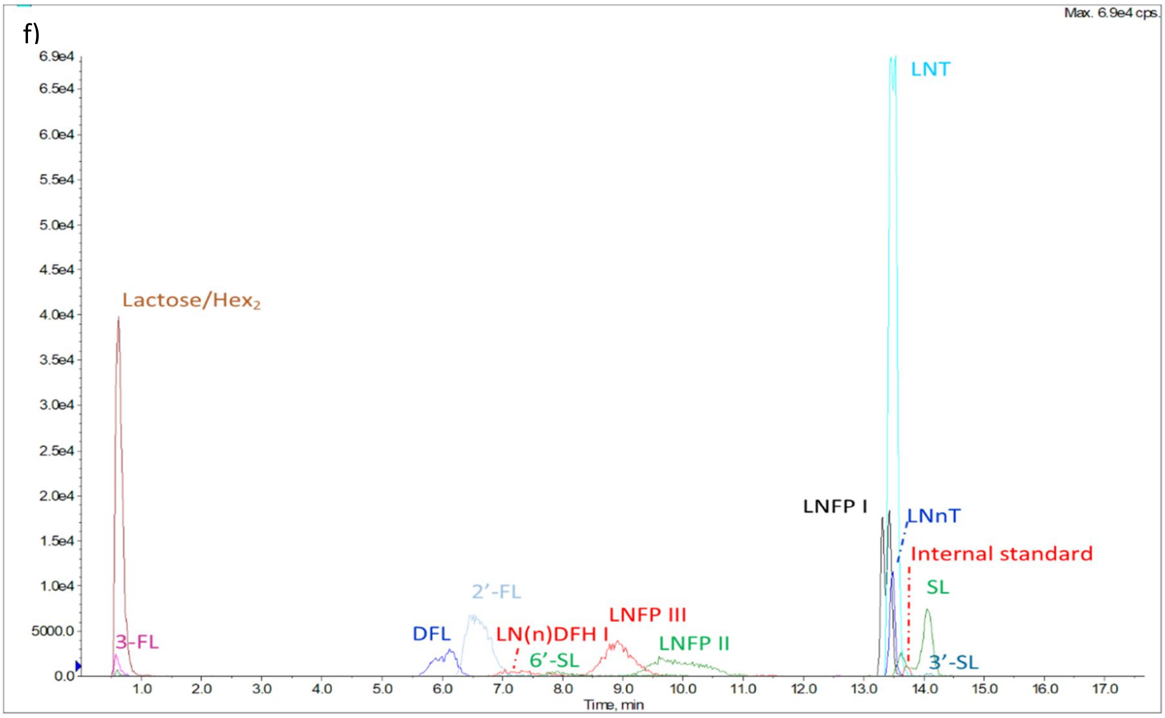
f)
Max. 6.9e4 cps.
LNT
Lactose/Hex2
LNFP I
LNnT
Internal standard
SL
2'-FL
LNFP III
DFL
LN(n)DFH I
LNFP II
6'-SL
3-FL
3'-SL
Time, min
